# Supplementary material for: Determination of the Roles of H. pylori Outer Membrane Virulence Factors and Pyroptosis-Associated NLRP3, ASC, Caspase-1, Gasdermin D, IL-1β, and IL-18 in Ulcer and Gastritis Pathogenesis
Source: Biology (Basel). 2025 May 30;14(6):634. doi: 10.3390/biology14060634 (PMC12189653; doi:10.3390/biology14060634)

Supplementary Figure S1. Graphs for gastritis patients that show the distribution of target markers by expression of virulence genes with upregulated pyroptosis markers. Error bars in all graphs represent the mean ± standard error of the mean (SEM), unless otherwise indicated.

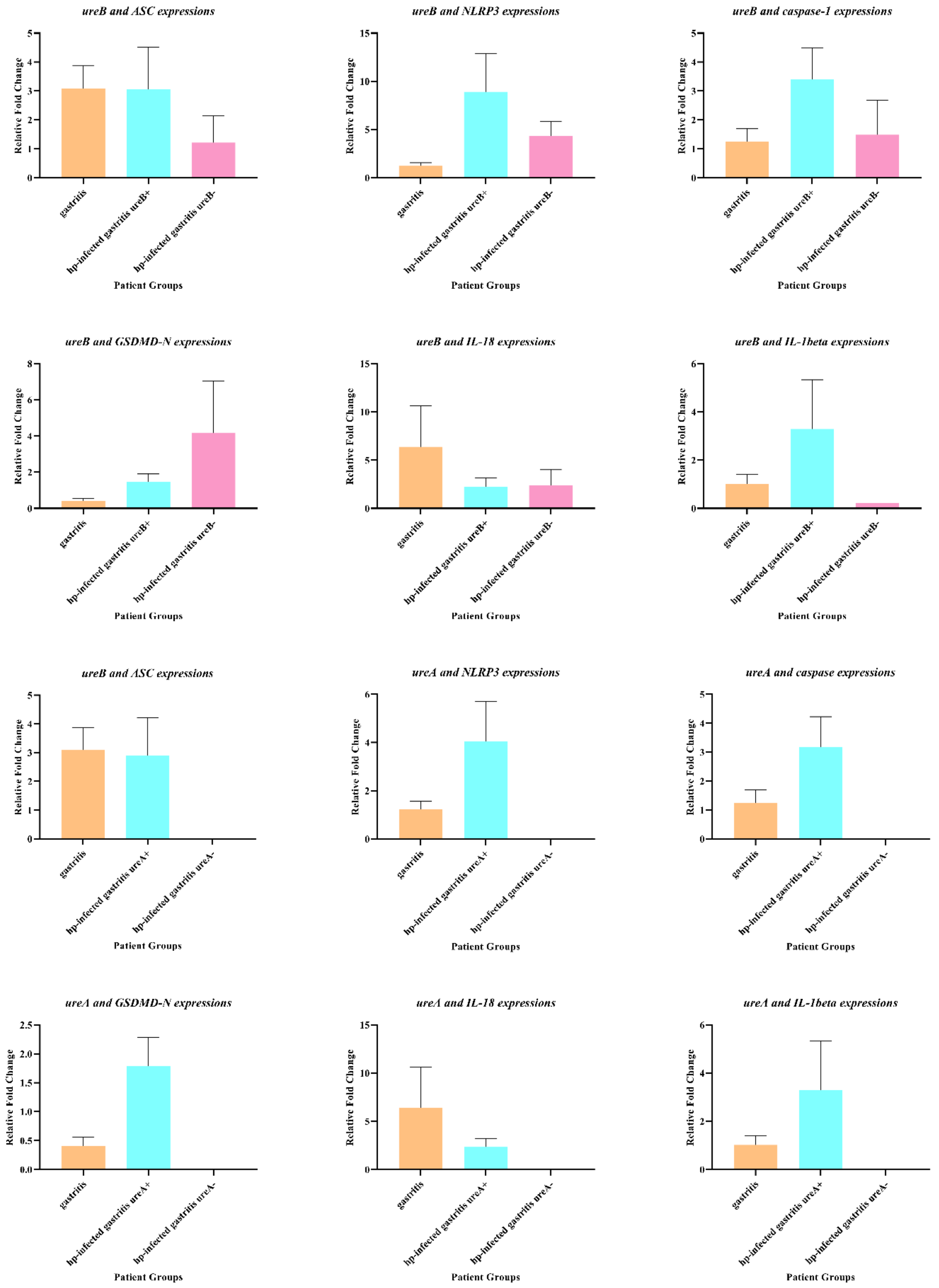

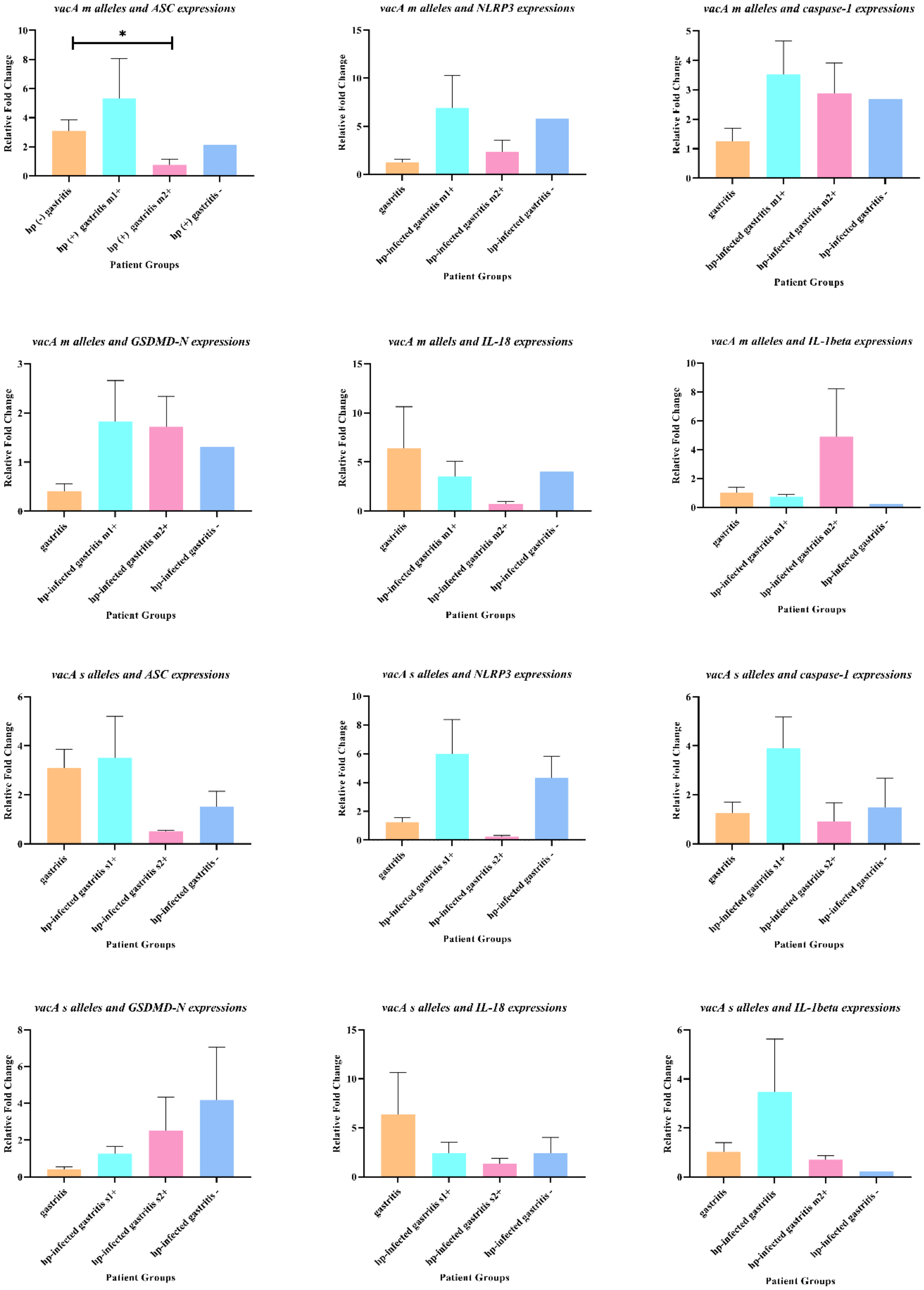

vacA m alleles and ASC expressions
Relative Fold Change
*
hp (-) gastritis
hp (+) gastritis m1+
hp (+) gastritis m2+
hp (+) gastritis -
Patient Groups
vacA m alleles and NLRP3 expressions
gastritis
hp-infected gastritis m1+
hp-infected gastritis m2+
hp-infected gastritis -
vacA m alleles and caspase-1 expressions
vacA m alleles and GSDMD-N expressions
vacA m allels and IL-18 expressions
vacA m alleles and IL-1beta expressions
vacA s alleles and ASC expressions
hp-infected gastritis s1+
hp-infected gastritis s2+
vacA s alleles and NLRP3 expressions
vacA s alleles and caspase-1 expressions
vacA s alleles and GSDMD-N expressions
vacA s alleles and IL-18 expressions
vacA s alleles and IL-1beta expressions
hp-infected gastritis

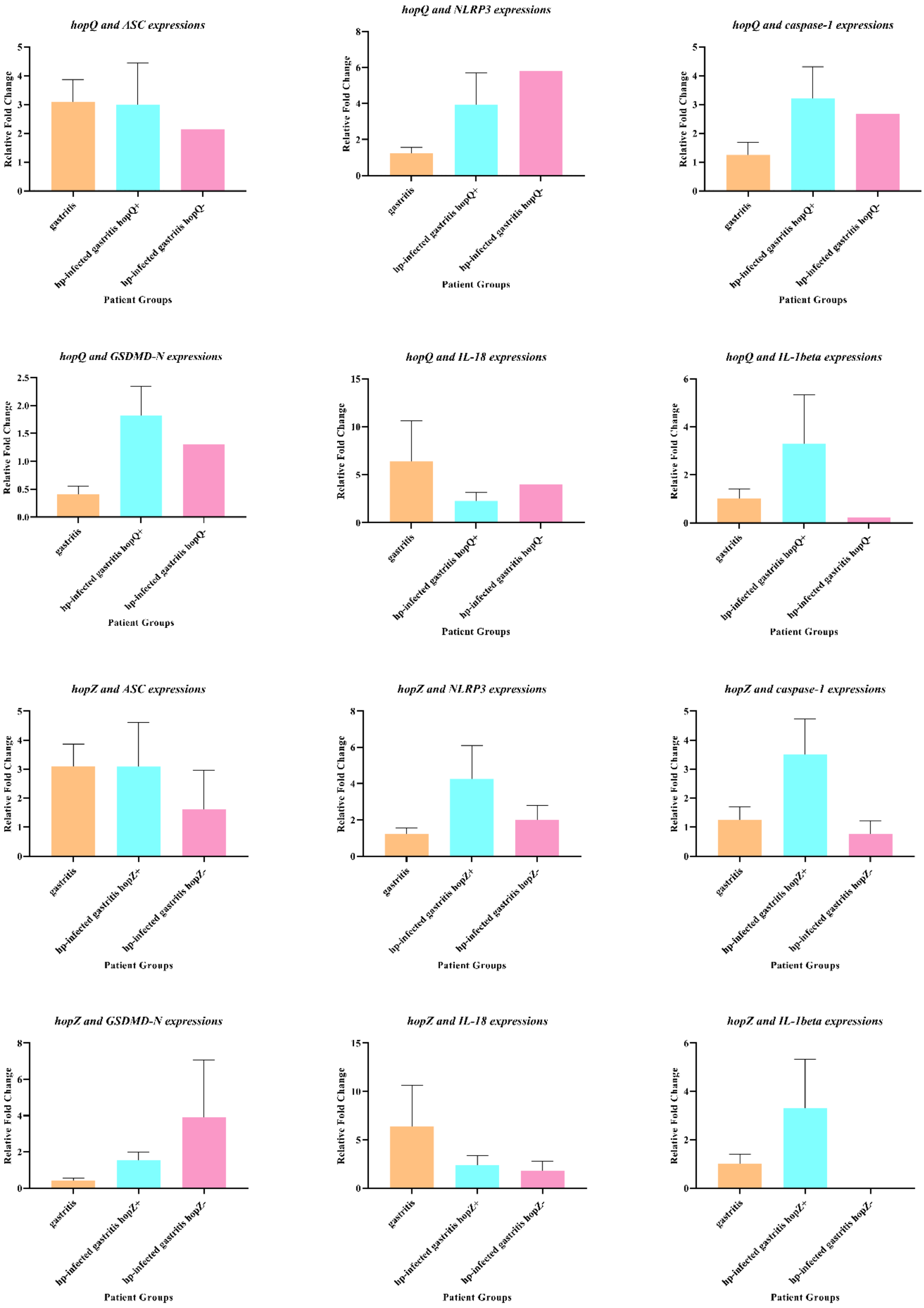
hopQ and ASC expressions
Relative Fold Change
0
1
2
3
4
5
gastritis
hp-infected gastritis hopQ+
hp-infected gastritis hopQ-
Patient Groups
hopQ and NLRP3 expressions
Relative Fold Change
0
2
4
6
8
gastritis
hp-infected gastritis hopQ+
hp-infected gastritis hopQ-
Patient Groups
hopQ and caspase-1 expressions
Relative Fold Change
0
1
2
3
4
5
gastritis
hp-infected gastritis hopQ+
hp-infected gastritis hopQ-
Patient Groups
hopQ and GSDMD-N expressions
Relative Fold Change
0.0
0.5
1.0
1.5
2.0
2.5
gastritis
hp-infected gastritis hopQ+
hp-infected gastritis hopQ-
Patient Groups
hopQ and IL-18 expressions
Relative Fold Change
0
5
10
15
gastritis
hp-infected gastritis hopQ+
hp-infected gastritis hopQ-
Patient Groups
hopQ and IL-1beta expressions
Relative Fold Change
0
2
4
6
gastritis
hp-infected gastritis hopQ+
hp-infected gastritis hopQ-
Patient Groups
hopZ and ASC expressions
Relative Fold Change
0
1
2
3
4
5
gastritis
hp-infected gastritis hopZ+
hp-infected gastritis hopZ-
Patient Groups
hopZ and NLRP3 expressions
Relative Fold Change
0
2
4
6
8
gastritis
hp-infected gastritis hopZ+
hp-infected gastritis hopZ-
Patient Groups
hopZ and caspase-1 expressions
Relative Fold Change
0
1
2
3
4
5
gastritis
hp-infected gastritis hopZ+
hp-infected gastritis hopZ-
Patient Groups
hopZ and GSDMD-N expressions
Relative Fold Change
0
2
4
6
8
gastritis
hp-infected gastritis hopZ+
hp-infected gastritis hopZ-
Patient Groups
hopZ and IL-18 expressions
Relative Fold Change
0
5
10
15
gastritis
hp-infected gastritis hopZ+
hp-infected gastritis hopZ-
Patient Groups
hopZ and IL-1beta expressions
Relative Fold Change
0
2
4
6
gastritis
hp-infected gastritis hopZ+
hp-infected gastritis hopZ-
Patient Groups

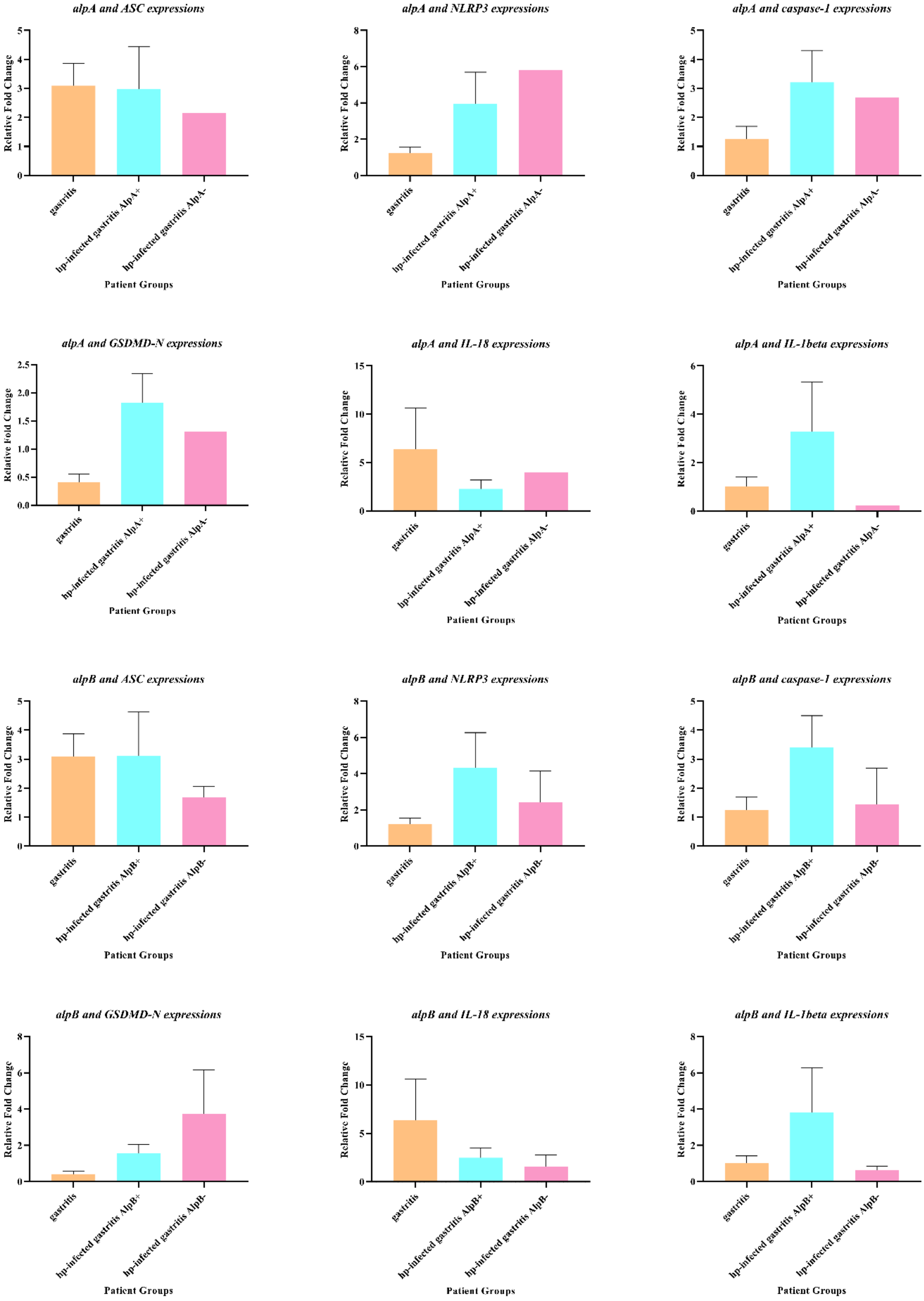
alpA and ASC expressions
alpA and NLRP3 expressions
alpA and caspase-1 expressions
alpA and GSDMD-N expressions
alpA and IL-18 expressions
alpA and IL-1beta expressions
alpB and ASC expressions
alpB and NLRP3 expressions
alpB and caspase-1 expressions
alpB and GSDMD-N expressions
alpB and IL-18 expressions
alpB and IL-1beta expressions
Relative Fold Change
gastritis
hp-infected gastritis AlpA+
hp-infected gastritis AlpA-
hp-infected gastritis AlpB+
hp-infected gastritis AlpB-
Patient Groups

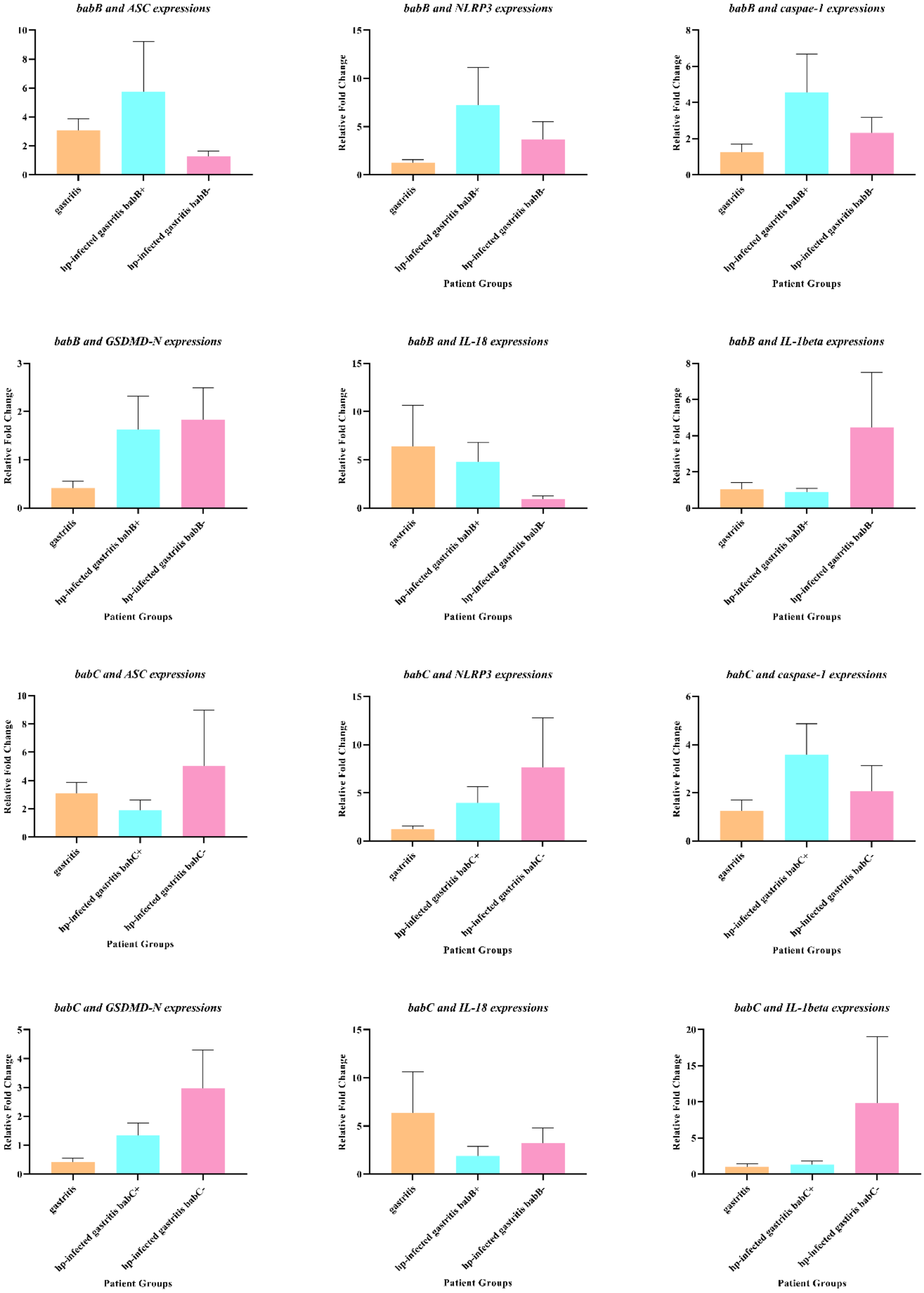

babB and ASC expressions
gastritis
hp-infected gastritis babB+
hp-infected gastritis babB-
babB and NLRP3 expressions
Relative Fold Change
gastritis
hp-infected gastritis babB+
hp-infected gastritis babB-
Patient Groups
babB and caspae-1 expressions
Relative Fold Change
gastritis
hp-infected gastritis babB+
hp-infected gastritis babB-
Patient Groups
babB and GSDMD-N expressions
Relative Fold Change
gastritis
hp-infected gastritis babB+
hp-infected gastritis babB-
Patient Groups
babB and IL-18 expressions
Relative Fold Change
gastritis
hp-infected gastritis babB+
hp-infected gastritis babB-
Patient Groups
babB and IL-1beta expressions
Relative Fold Change
gastritis
hp-infected gastritis babB+
hp-infected gastritis babB-
Patient Groups
babC and ASC expressions
Relative Fold Change
gastritis
hp-infected gastritis babC+
hp-infected gastritis babC-
Patient Groups
babC and NLRP3 expressions
Relative Fold Change
gastritis
hp-infected gastritis babC+
hp-infected gastritis babC-
Patient Groups
babC and caspase-1 expressions
Relative Fold Change
gastritis
hp-infected gastritis babC+
hp-infected gastritis babC-
Patient Groups
babC and GSDMD-N expressions
Relative Fold Change
gastritis
hp-infected gastritis babC+
hp-infected gastritis babC-
Patient Groups
babC and IL-18 expressions
Relative Fold Change
gastritis
hp-infected gastritis babB+
hp-infected gastritis babB-
Patient Groups
babC and IL-1beta expressions
Relative Fold Change
gastritis
hp-infected gastritis babC+
hp-infected gastritis babC-
Patient Groups

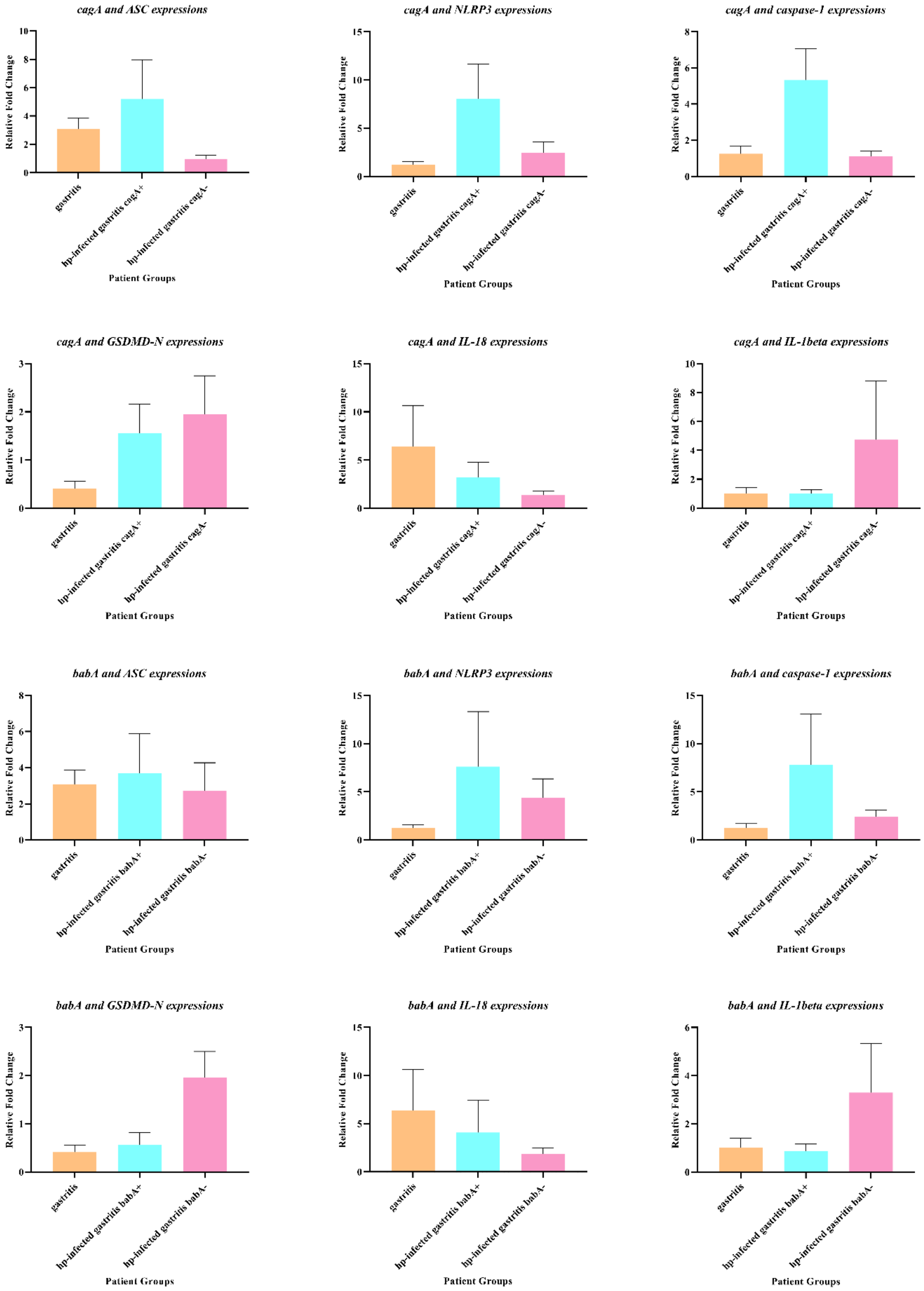
cagA and ASC expressions
cagA and NLRP3 expressions
cagA and caspase-1 expressions
cagA and GSDMD-N expressions
cagA and IL-18 expressions
cagA and IL-1beta expressions
babA and ASC expressions
babA and NLRP3 expressions
babA and caspase-1 expressions
babA and GSDMD-N expressions
babA and IL-18 expressions
babA and IL-1beta expressions
Relative Fold Change
Patient Groups
gastritis
hp-infected gastritis cagA+
hp-infected gastritis cagA-
hp-infected gastritis babA+
hp-infected gastritis babA-

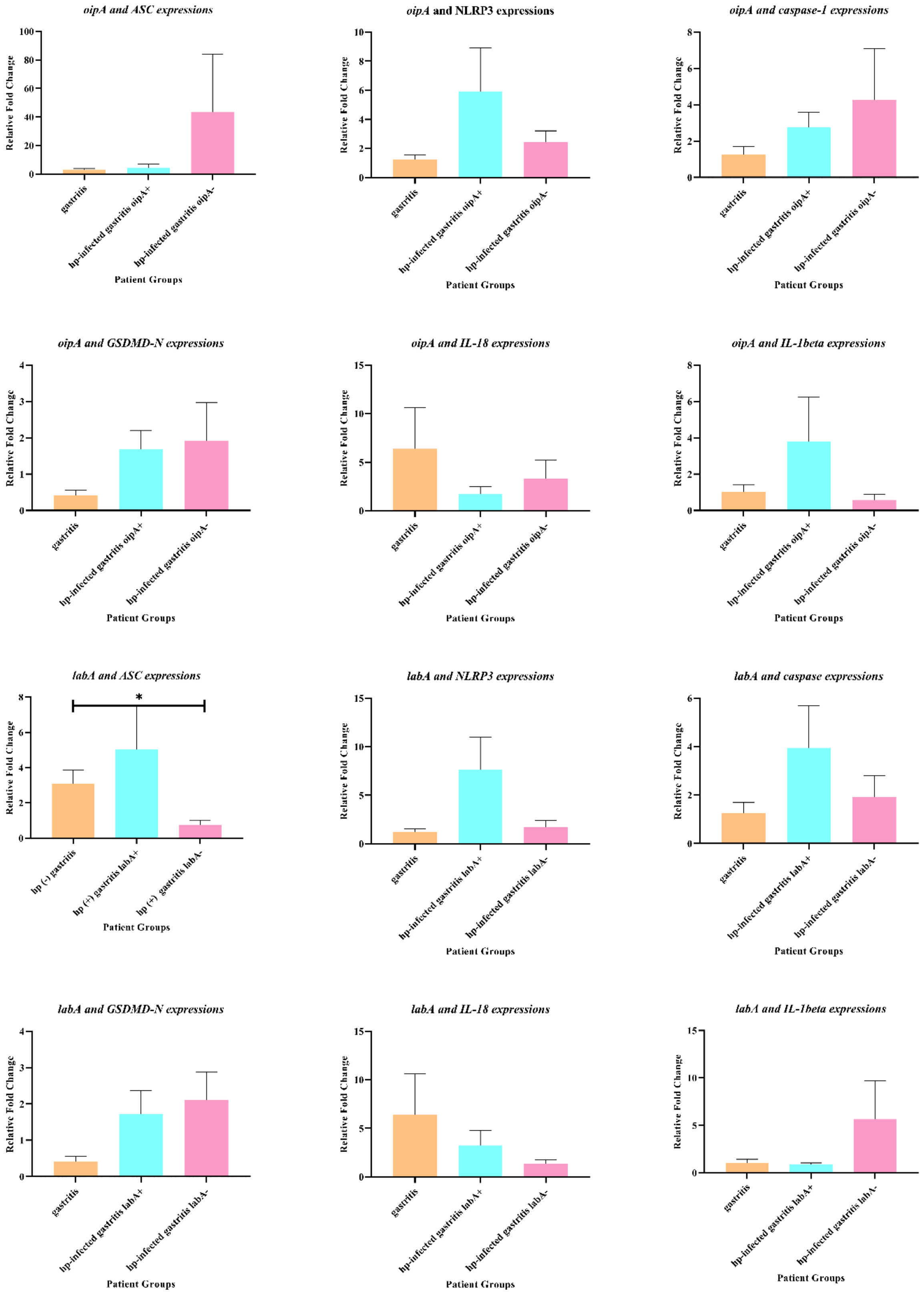
oipA and ASC expressions
Relative Fold Change
100
80
60
40
20
0
gastritis
hp-infected gastritis oipA+
hp-infected gastritis oipA-
Patient Groups
oipA and NLRP3 expressions
Relative Fold Change
10
8
6
4
2
0
gastritis
hp-infected gastritis oipA+
hp-infected gastritis oipA-
Patient Groups
oipA and caspase-1 expressions
Relative Fold Change
8
6
4
2
0
gastritis
hp-infected gastritis oipA+
hp-infected gastritis oipA-
Patient Groups
oipA and GSDMD-N expressions
Relative Fold Change
4
3
2
1
0
gastritis
hp-infected gastritis oipA+
hp-infected gastritis oipA-
Patient Groups
oipA and IL-18 expressions
Relative Fold Change
15
10
5
0
gastritis
hp-infected gastritis oipA+
hp-infected gastritis oipA-
Patient Groups
oipA and IL-1beta expressions
Relative Fold Change
8
6
4
2
0
gastritis
hp-infected gastritis oipA+
hp-infected gastritis oipA-
Patient Groups
labA and ASC expressions
Relative Fold Change
*
8
6
4
2
0
hp (-) gastritis
hp (+) gastritis labA+
hp (+) gastritis labA-
Patient Groups
labA and NLRP3 expressions
Relative Fold Change
15
10
5
0
gastritis
hp-infected gastritis labA+
hp-infected gastritis labA-
Patient Groups
labA and caspase expressions
Relative Fold Change
6
4
2
0
gastritis
hp-infected gastritis labA+
hp-infected gastritis labA-
Patient Groups
labA and GSDMD-N expressions
Relative Fold Change
4
3
2
1
0
gastritis
hp-infected gastritis labA+
hp-infected gastritis labA-
Patient Groups
labA and IL-18 expressions
Relative Fold Change
15
10
5
0
gastritis
hp-infected gastritis labA+
hp-infected gastritis labA-
Patient Groups
labA and IL-1beta expressions
Relative Fold Change
15
10
5
0
gastritis
hp-infected gastritis labA+
hp-infected gastritis labA-
Patient Groups

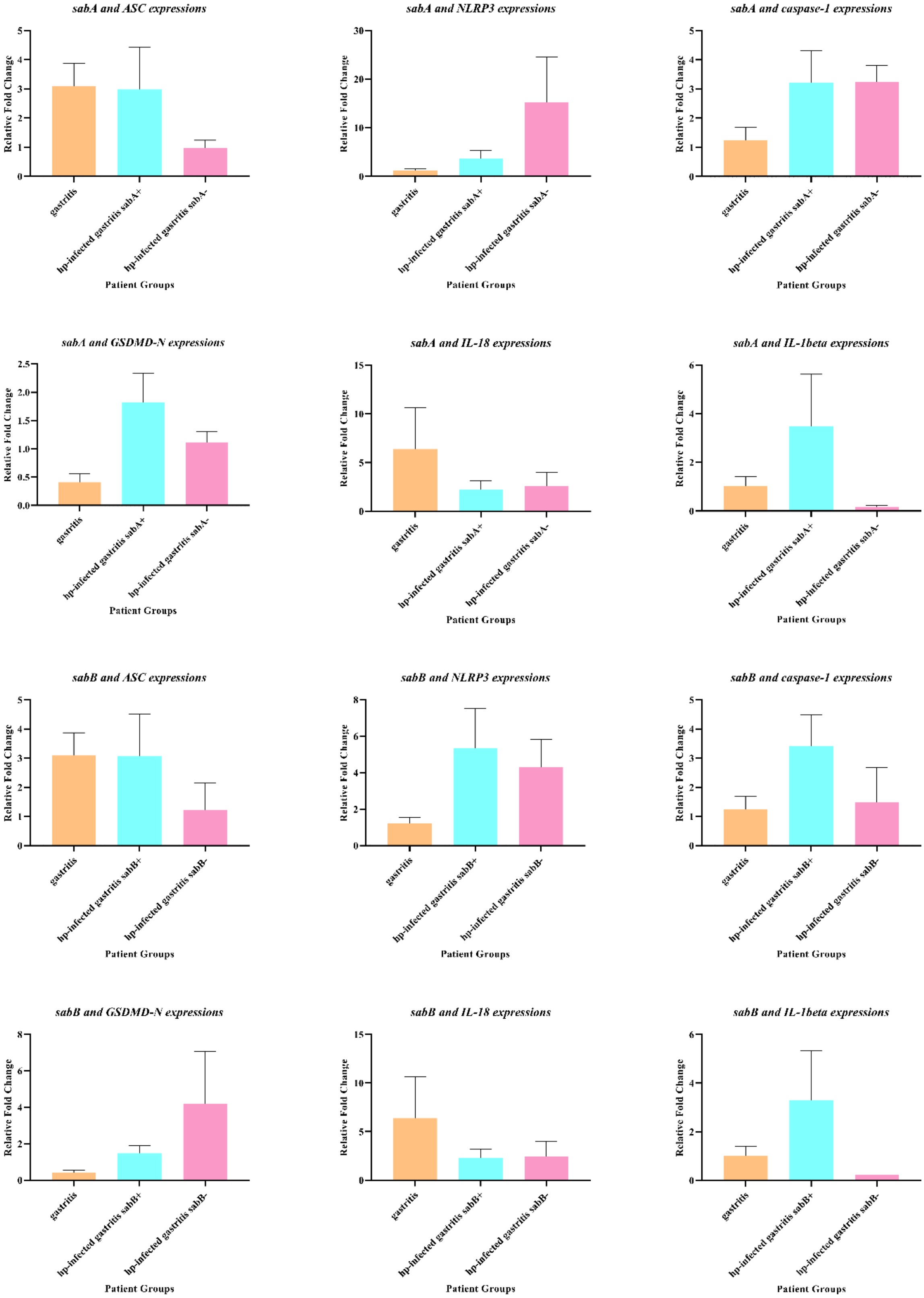
sabA and ASC expressions
sabA and NLRP3 expressions
sabA and caspase-1 expressions
sabA and GSDMD-N expressions
sabA and IL-18 expressions
sabA and IL-1beta expressions
sabB and ASC expressions
sabB and NLRP3 expressions
sabB and caspase-1 expressions
sabB and GSDMD-N expressions
sabB and IL-18 expressions
sabB and IL-1beta expressions
Relative Fold Change
gastritis
hp-infected gastritis sabA+
hp-infected gastritis sabA-
hp-infected gastritis sabB+
hp-infected gastritis sabB-
Patient Groups

Supplementary Figure S2. Graphs showing the change in pyroptosis response in the presence of bacterial virulence genes in infected ulcer patients. Error bars in all graphs represent the mean ± standard error of the mean (SEM), unless otherwise indicated.

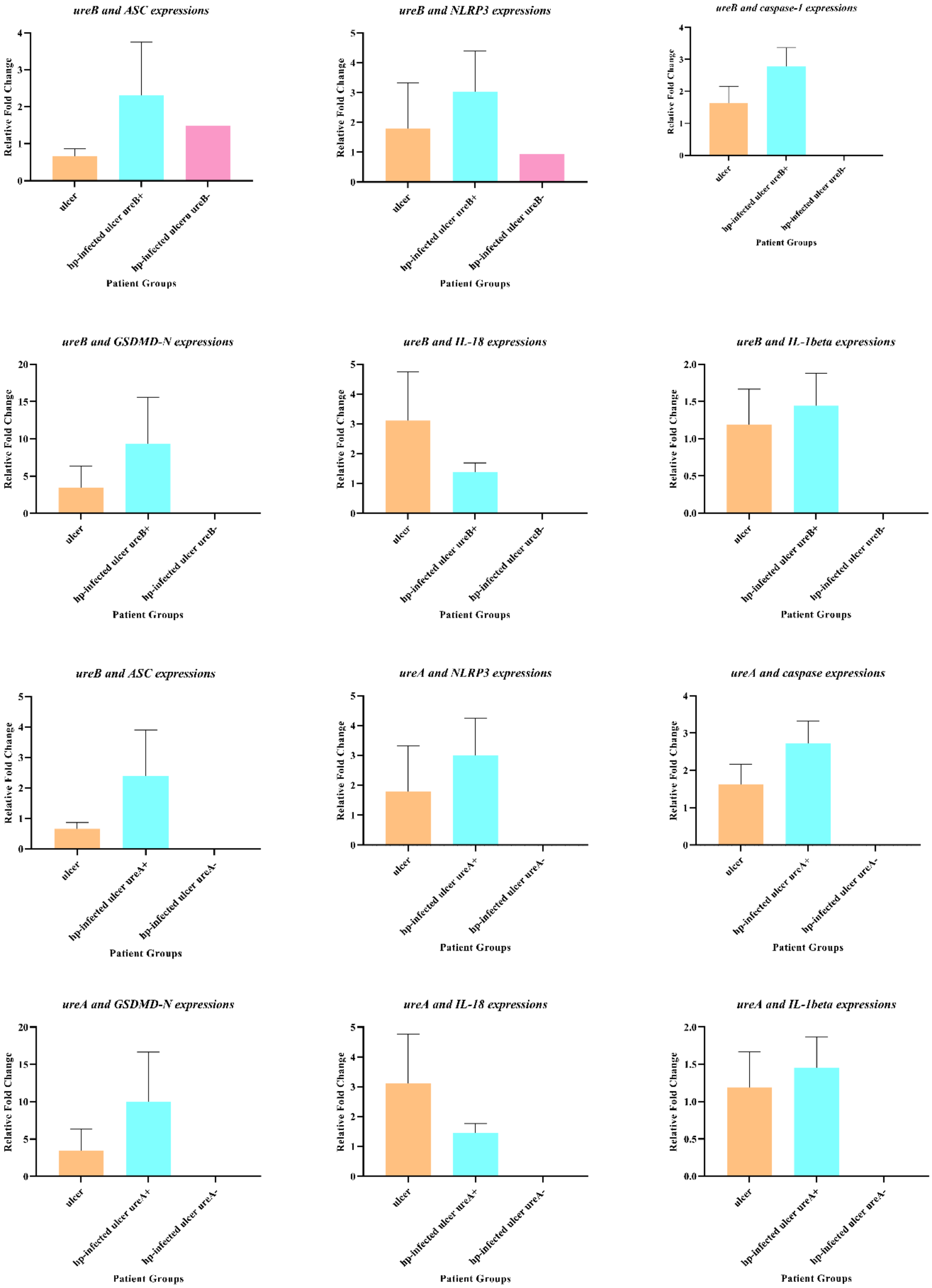
ureB and ASC expressions
Relative Fold Change
0
1
2
3
4
ulcer
hp-infected ulcer ureB+
hp-infected ulceru ureB-
Patient Groups
ureB and NLRP3 expressions
Relative Fold Change
0
1
2
3
4
5
ulcer
hp-infected ulcer ureB+
hp-infected ulcer ureB-
Patient Groups
ureB and caspase-1 expressions
Relative Fold Change
0
1
2
3
4
ulcer
hp-infected ulcer ureB+
hp-infected ulcer ureB-
Patient Groups
ureB and GSDMD-N expressions
Relative Fold Change
0
5
10
15
20
ulcer
hp-infected ulcer ureB+
hp-infected ulcer ureB-
Patient Groups
ureB and IL-18 expressions
Relative Fold Change
0
1
2
3
4
5
ulcer
hp-infected ulcer ureB+
hp-infected ulcer ureB-
Patient Groups
ureB and IL-1beta expressions
Relative Fold Change
0.0
0.5
1.0
1.5
2.0
ulcer
hp-infected ulcer ureB+
hp-infected ulcer ureB-
Patient Groups
ureB and ASC expressions
Relative Fold Change
0
1
2
3
4
5
ulcer
hp-infected ulcer ureA+
hp-infected ulcer ureA-
Patient Groups
ureA and NLRP3 expressions
Relative Fold Change
0
1
2
3
4
5
ulcer
hp-infected ulcer ureA+
hp-infected ulcer ureA-
Patient Groups
ureA and caspase expressions
Relative Fold Change
0
1
2
3
4
ulcer
hp-infected ulcer ureA+
hp-infected ulcer ureA-
Patient Groups
ureA and GSDMD-N expressions
Relative Fold Change
0
5
10
15
20
ulcer
hp-infected ulcer ureA+
hp-infected ulcer ureA-
Patient Groups
ureA and IL-18 expressions
Relative Fold Change
0
1
2
3
4
5
ulcer
hp-infected ulcer ureA+
hp-infected ulcer ureA-
Patient Groups
ureA and IL-1beta expressions
Relative Fold Change
0.0
0.5
1.0
1.5
2.0
ulcer
hp-infected ulcer ureA+
hp-infected ulcer ureA-
Patient Groups

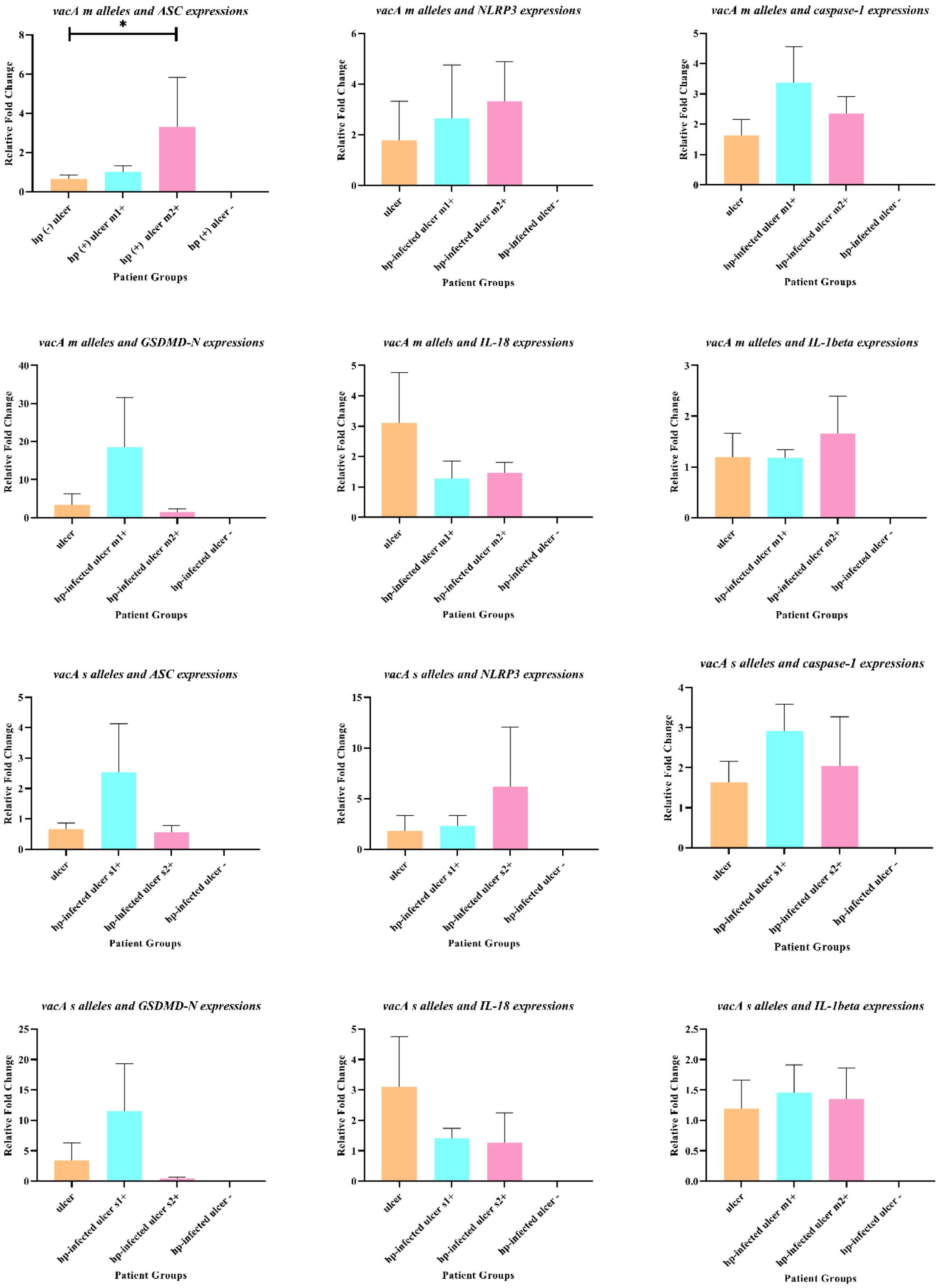
vacA m alleles and ASC expressions
*
Relative Fold Change
hp (-) ulcer
hp (+) ulcer m1+
hp (+) ulcer m2+
hp (+) ulcer -
Patient Groups
vacA m alleles and NLRP3 expressions
Relative Fold Change
ulcer
hp-infected ulcer m1+
hp-infected ulcer m2+
hp-infected ulcer -
Patient Groups
vacA m alleles and caspase-1 expressions
Relative Fold Change
ulcer
hp-infected ulcer m1+
hp-infected ulcer m2+
hp-infected ulcer -
Patient Groups
vacA m alleles and GSDMD-N expressions
Relative Fold Change
ulcer
hp-infected ulcer m1+
hp-infected ulcer m2+
hp-infected ulcer -
Patient Groups
vacA m allels and IL-18 expressions
Relative Fold Change
ulcer
hp-infected ulcer m1+
hp-infected ulcer m2+
hp-infected ulcer -
Patient Groups
vacA m alleles and IL-1beta expressions
Relative Fold Change
ulcer
hp-infected ulcer m1+
hp-infected ulcer m2+
hp-infected ulcer -
Patient Groups
vacA s alleles and ASC expressions
Relative Fold Change
ulcer
hp-infected ulcer s1+
hp-infected ulcer s2+
hp-infected ulcer -
Patient Groups
vacA s alleles and NLRP3 expressions
Relative Fold Change
ulcer
hp-infected ulcer s1+
hp-infected ulcer s2+
hp-infected ulcer -
Patient Groups
vacA s alleles and caspase-1 expressions
Relative Fold Change
ulcer
hp-infected ulcer s1+
hp-infected ulcer s2+
hp-infected ulcer -
Patient Groups
vacA s alleles and GSDMD-N expressions
Relative Fold Change
ulcer
hp-infected ulcer s1+
hp-infected ulcer s2+
hp-infected ulcer -
Patient Groups
vacA s alleles and IL-18 expressions
Relative Fold Change
ulcer
hp-infected ulcer s1+
hp-infected ulcer s2+
hp-infected ulcer -
Patient Groups
vacA s alleles and IL-1beta expressions
Relative Fold Change
ulcer
hp-infected ulcer m1+
hp-infected ulcer m2+
hp-infected ulcer -
Patient Groups

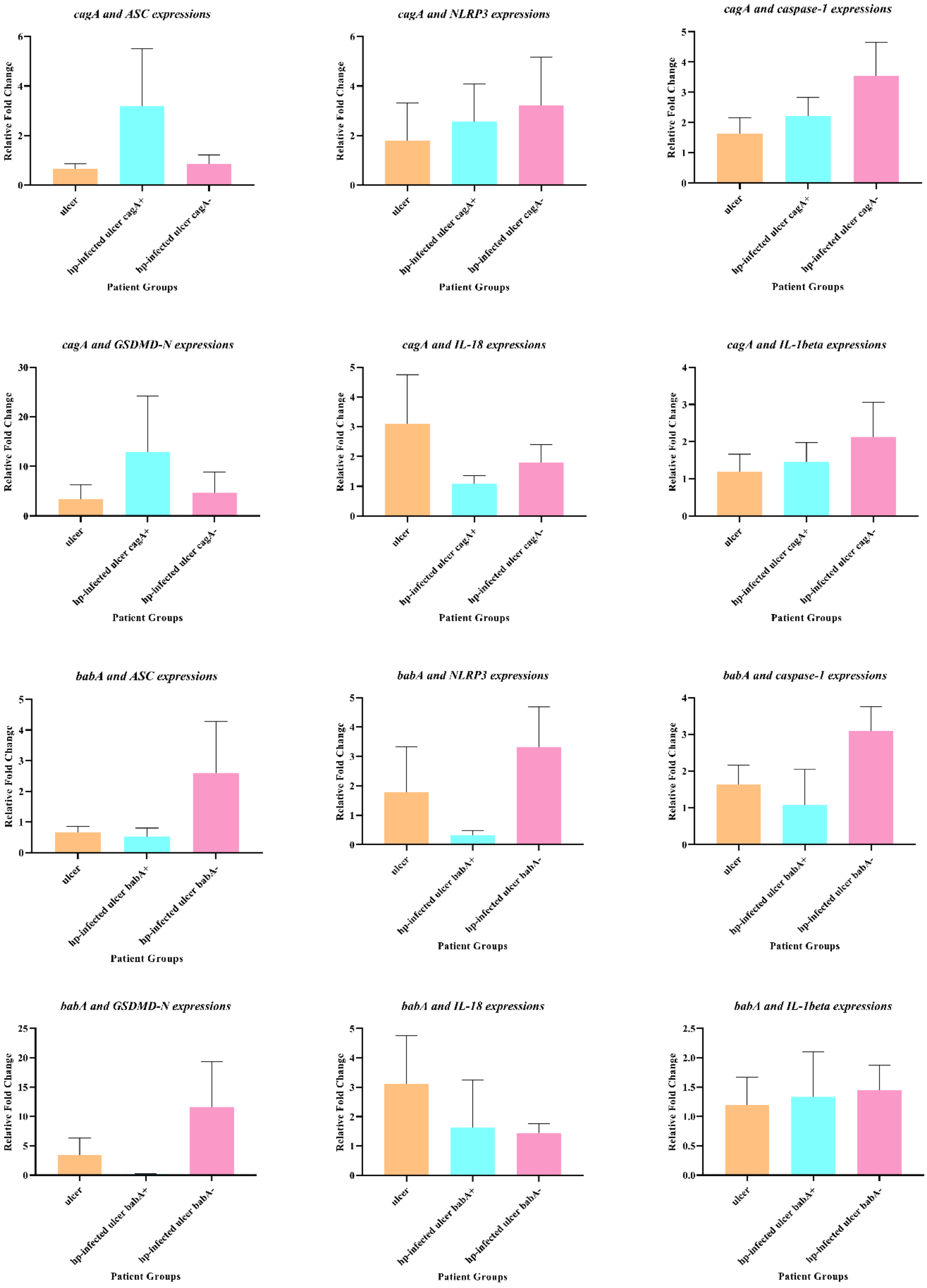
cagA and ASC expressions
cagA and NLRP3 expressions
cagA and caspase-1 expressions
cagA and GSDMD-N expressions
cagA and IL-18 expressions
cagA and IL-1beta expressions
babA and ASC expressions
babA and NLRP3 expressions
babA and caspase-1 expressions
babA and GSDMD-N expressions
babA and IL-18 expressions
babA and IL-1beta expressions
Relative Fold Change
Patient Groups
ulcer
hp-infected ulcer cagA+
hp-infected ulcer cagA-
hp-infected ulcer babA+
hp-infected ulcer babA-

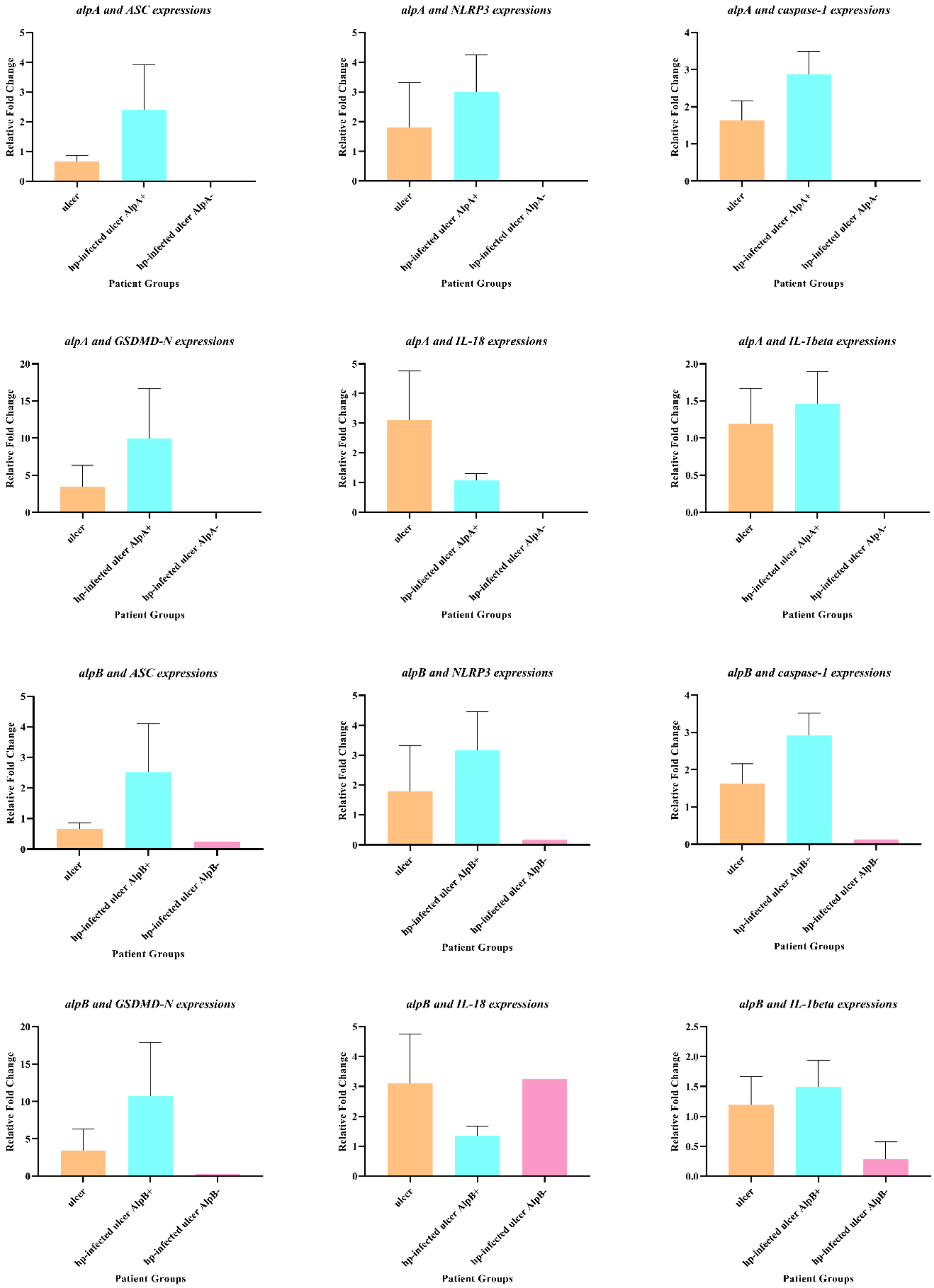

alpA and ASC expressions
alpA and NLRP3 expressions
alpA and caspase-1 expressions
alpA and GSDMD-N expressions
alpA and IL-18 expressions
alpA and IL-1beta expressions
alpB and ASC expressions
alpB and NLRP3 expressions
alpB and caspase-1 expressions
alpB and GSDMD-N expressions
alpB and IL-18 expressions
alpB and IL-1beta expressions
Relative Fold Change
Patient Groups
ulcer
hp-infected ulcer AlpA+
hp-infected ulcer AlpA-
hp-infected ulcer AlpB+
hp-infected ulcer AlpB-

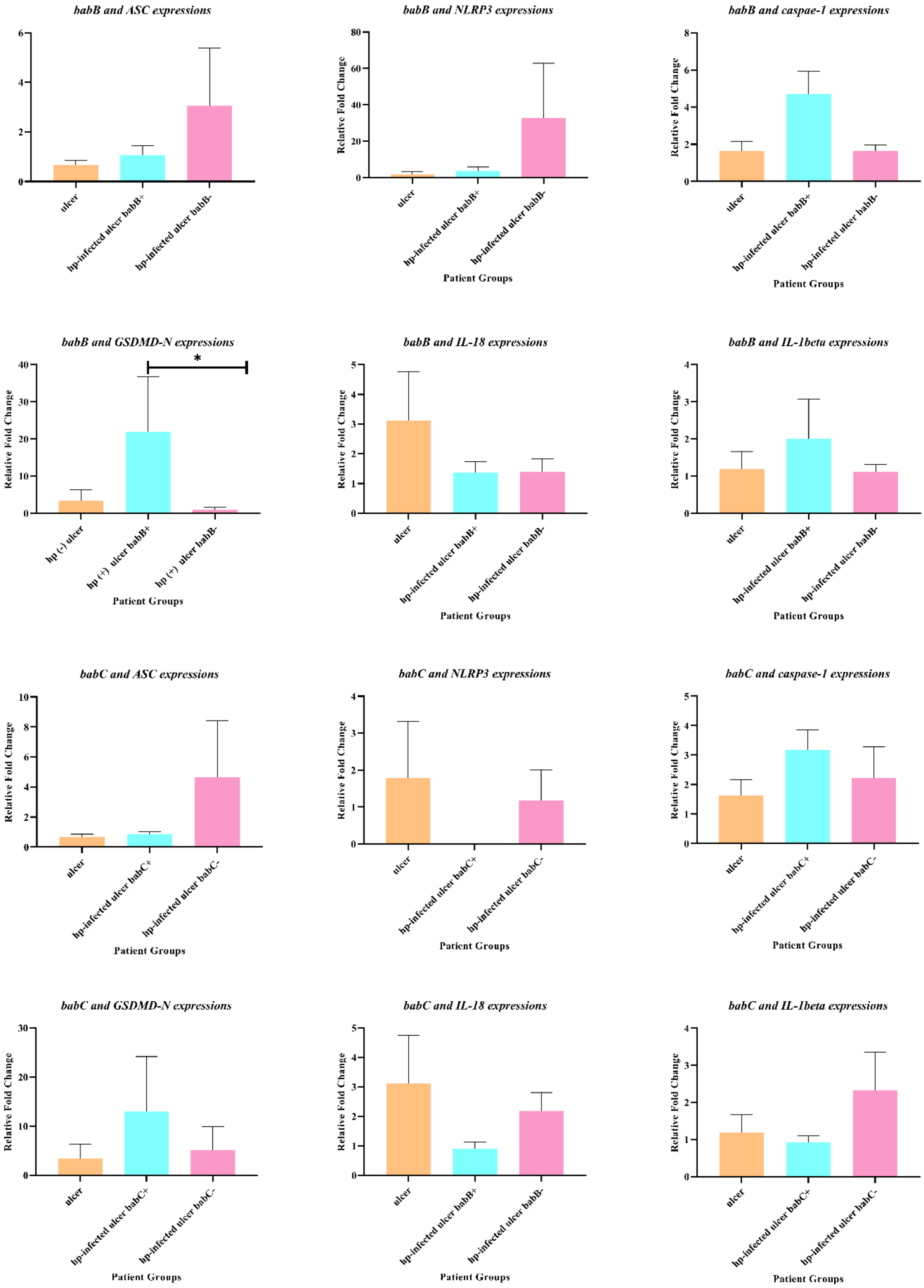
babB and ASC expressions
0
2
4
6
ulcer
hp-infected ulcer babB+
hp-infected ulcer babB-
babB and NLRP3 expressions
Relative Fold Change
0
20
40
60
80
ulcer
hp-infected ulcer babB+
hp-infected ulcer babB-
Patient Groups
babB and caspae-1 expressions
Relative Fold Change
0
2
4
6
8
ulcer
hp-infected ulcer babB+
hp-infected ulcer babB-
Patient Groups
babB and GSDMD-N expressions
Relative Fold Change
0
10
20
30
40
*
hp (-) ulcer
hp (+) ulcer babB+
hp (+) ulcer babB-
Patient Groups
babB and IL-18 expressions
Relative Fold Change
0
1
2
3
4
5
ulcer
hp-infected ulcer babB+
hp-infected ulcer babB-
Patient Groups
babB and IL-1beta expressions
Relative Fold Change
0
1
2
3
4
ulcer
hp-infected ulcer babB+
hp-infected ulcer babB-
Patient Groups
babC and ASC expressions
Relative Fold Change
0
2
4
6
8
10
ulcer
hp-infected ulcer babC+
hp-infected ulcer babC-
Patient Groups
babC and NLRP3 expressions
Relative Fold Change
0
1
2
3
4
ulcer
hp-infected ulcer babC+
hp-infected ulcer babC-
Patient Groups
babC and caspase-1 expressions
Relative Fold Change
0
1
2
3
4
5
ulcer
hp-infected ulcer babC+
hp-infected ulcer babC-
Patient Groups
babC and GSDMD-N expressions
Relative Fold Change
0
10
20
30
ulcer
hp-infected ulcer babC+
hp-infected ulcer babC-
Patient Groups
babC and IL-18 expressions
Relative Fold Change
0
1
2
3
4
5
ulcer
hp-infected ulcer babB+
hp-infected ulcer babB-
Patient Groups
babC and IL-1beta expressions
Relative Fold Change
0
1
2
3
4
ulcer
hp-infected ulcer babC+
hp-infected ulcer babC-
Patient Groups

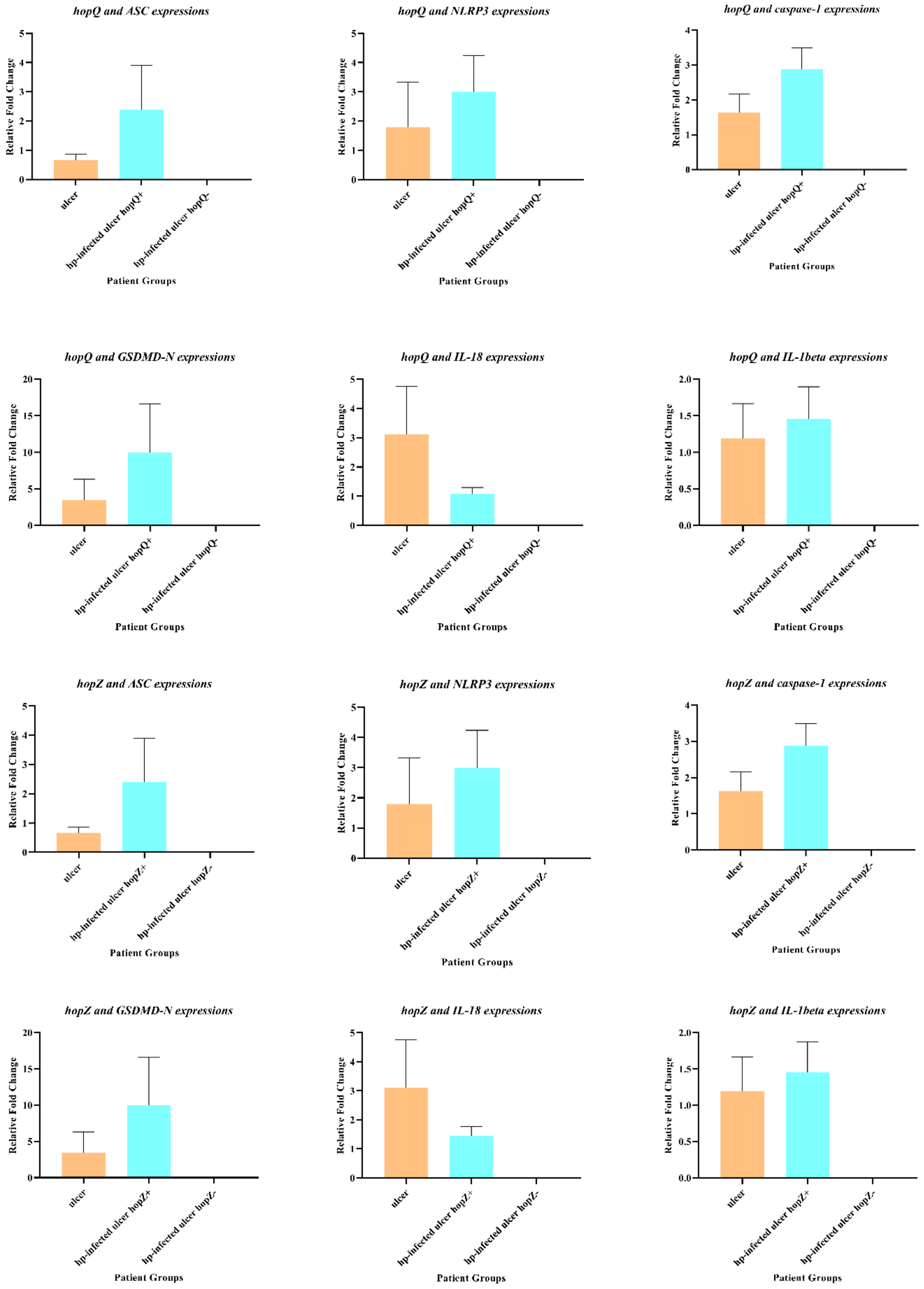
hopQ and ASC expressions
hopQ and NLRP3 expressions
hopQ and caspase-1 expressions
hopQ and GSDMD-N expressions
hopQ and IL-18 expressions
hopQ and IL-1beta expressions
hopZ and ASC expressions
hopZ and NLRP3 expressions
hopZ and caspase-1 expressions
hopZ and GSDMD-N expressions
hopZ and IL-18 expressions
hopZ and IL-1beta expressions
Relative Fold Change
Patient Groups
ulcer
hp-infected ulcer hopQ+
hp-infected ulcer hopQ-
hp-infected ulcer hopZ+
hp-infected ulcer hopZ-

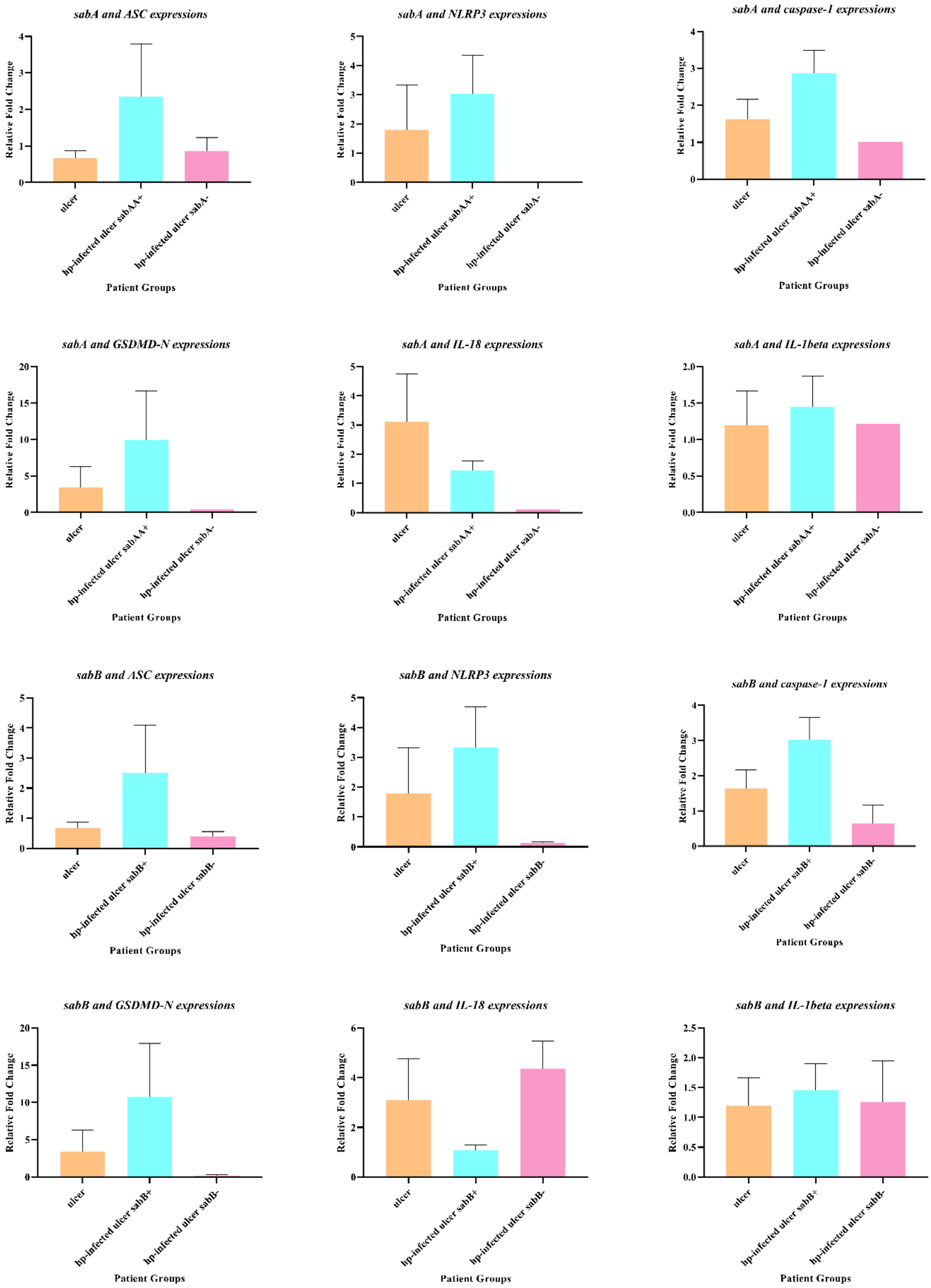

sabA and ASC expressions
Relative Fold Change
ulcer
hp-infected ulcer sabAA+
hp-infected ulcer sabA-
Patient Groups
sabA and NLRP3 expressions
Relative Fold Change
ulcer
hp-infected ulcer sabAA+
hp-infected ulcer sabA-
Patient Groups
sabA and caspase-1 expressions
Relative Fold Change
ulcer
hp-infected ulcer sabAA+
hp-infected ulcer sabA-
Patient Groups
sabA and GSDMD-N expressions
Relative Fold Change
ulcer
hp-infected ulcer sabAA+
hp-infected ulcer sabA-
Patient Groups
sabA and IL-18 expressions
Relative Fold Change
ulcer
hp-infected ulcer sabAA+
hp-infected ulcer sabA-
Patient Groups
sabA and IL-1beta expressions
Relative Fold Change
ulcer
hp-infected ulcer sabAA+
hp-infected ulcer sabA-
Patient Groups
sabB and ASC expressions
Relative Fold Change
ulcer
hp-infected ulcer sabB+
hp-infected ulcer sabB-
Patient Groups
sabB and NLRP3 expressions
Relative Fold Change
ulcer
hp-infected ulcer sabB+
hp-infected ulcer sabB-
Patient Groups
sabB and caspase-1 expressions
Relative Fold Change
ulcer
hp-infected ulcer sabB+
hp-infected ulcer sabB-
Patient Groups
sabB and GSDMD-N expressions
Relative Fold Change
ulcer
hp-infected ulcer sabB+
hp-infected ulcer sabB-
Patient Groups
sabB and IL-18 expressions
Relative Fold Change
ulcer
hp-infected ulcer sabB+
hp-infected ulcer sabB-
Patient Groups
sabB and IL-1beta expressions
Relative Fold Change
ulcer
hp-infected ulcer sabB+
hp-infected ulcer sabB-
Patient Groups

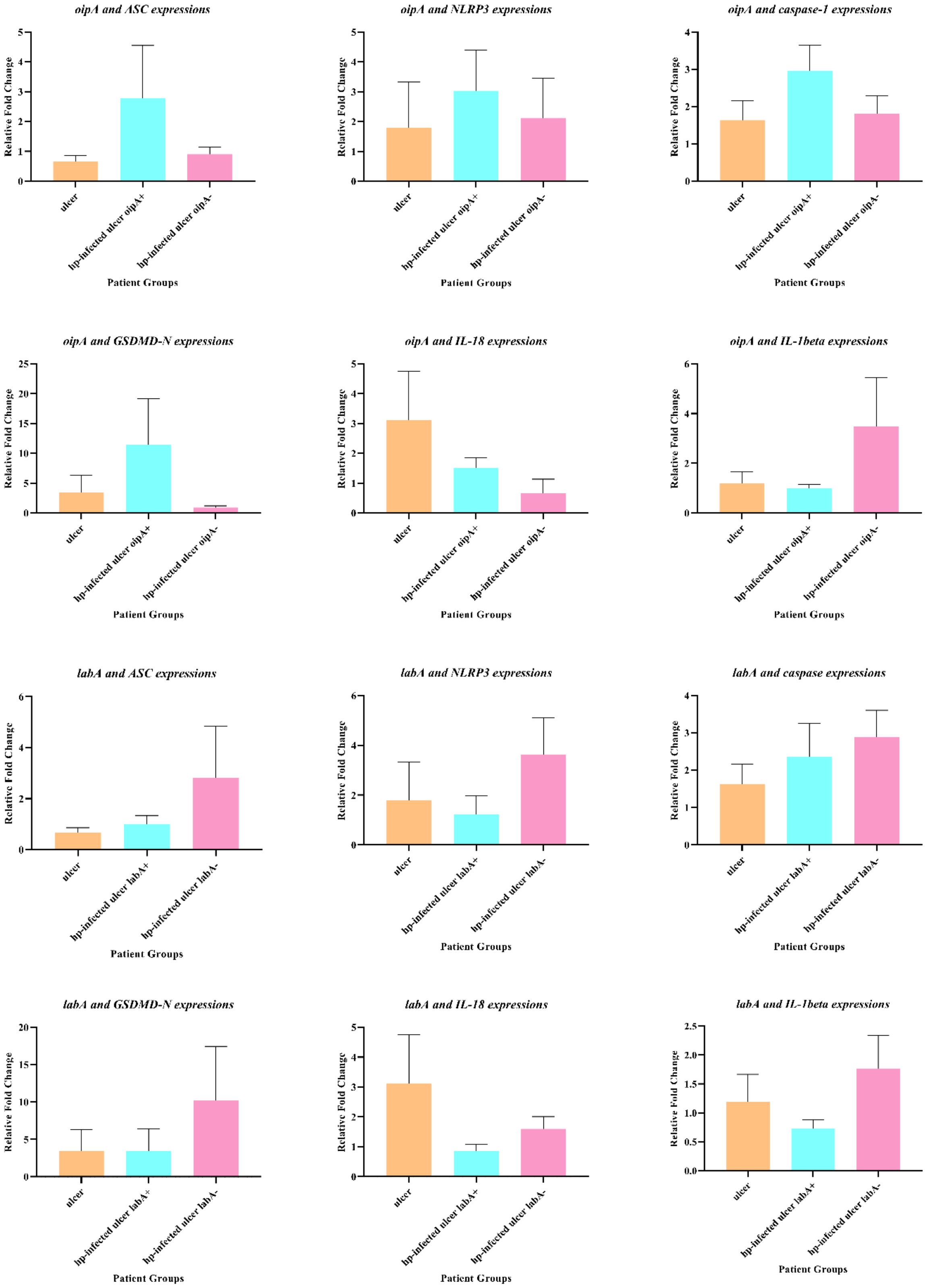
oipA and ASC expressions
oipA and NLRP3 expressions
oipA and caspase-1 expressions
oipA and GSDMD-N expressions
oipA and IL-18 expressions
oipA and IL-1beta expressions
labA and ASC expressions
labA and NLRP3 expressions
labA and caspase expressions
labA and GSDMD-N expressions
labA and IL-18 expressions
labA and IL-1beta expressions
Relative Fold Change
ulcer
hp-infected ulcer oipA+
hp-infected ulcer oipA-
hp-infected ulcer labA+
hp-infected ulcer labA-
Patient Groups

Supplementary Figure S3. Heat map analyses for the distribution of the number of all patients according to virulence genes and pyroptosis markers with infection severity

A.

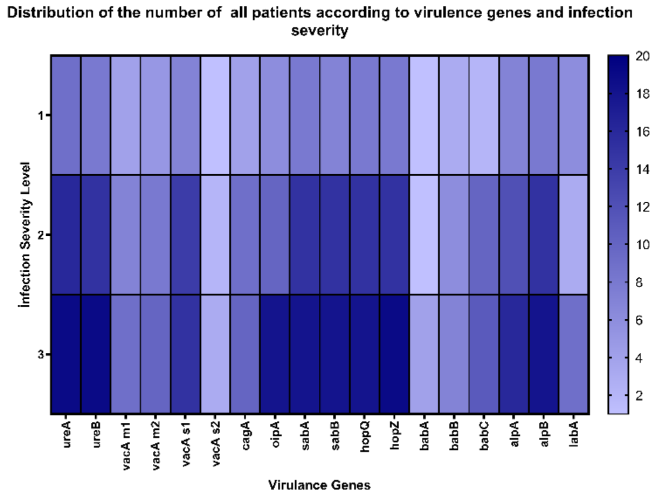

B.

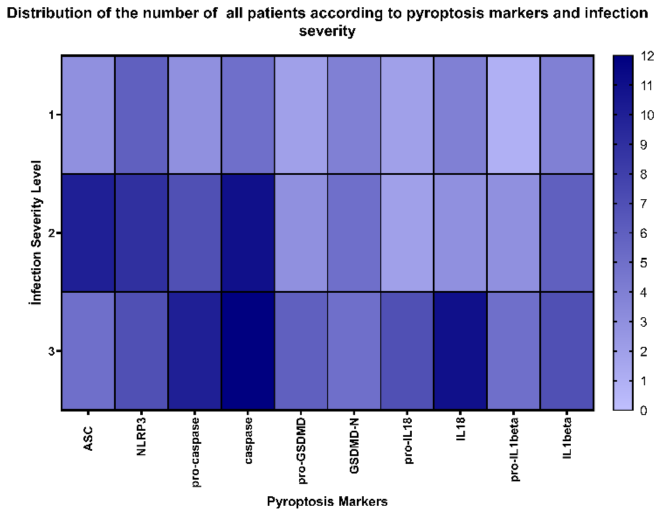

Supplementary Figure S4. Graphs for gastritis patients that show the severity of gastric inflammation and ulceration based on histopathological scoring and include correlation analyses between severity scores and gene expression levels. Error bars in all graphs represent the mean ± standard error of the mean (SEM), unless otherwise indicated.

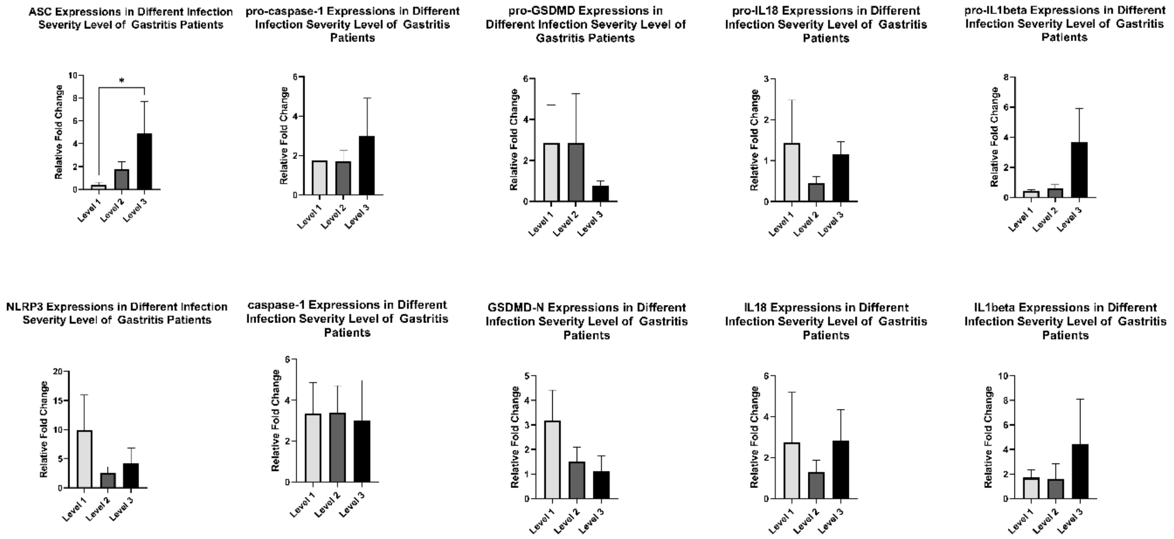

Supplementary Figure S5. Graphs for ulcer patients that show the severity of gastric inflammation and ulceration based on histopathological scoring and include correlation analyses between severity scores and gene expression levels. Error bars in all graphs represent the mean ± standard error of the mean (SEM), unless otherwise indicated.

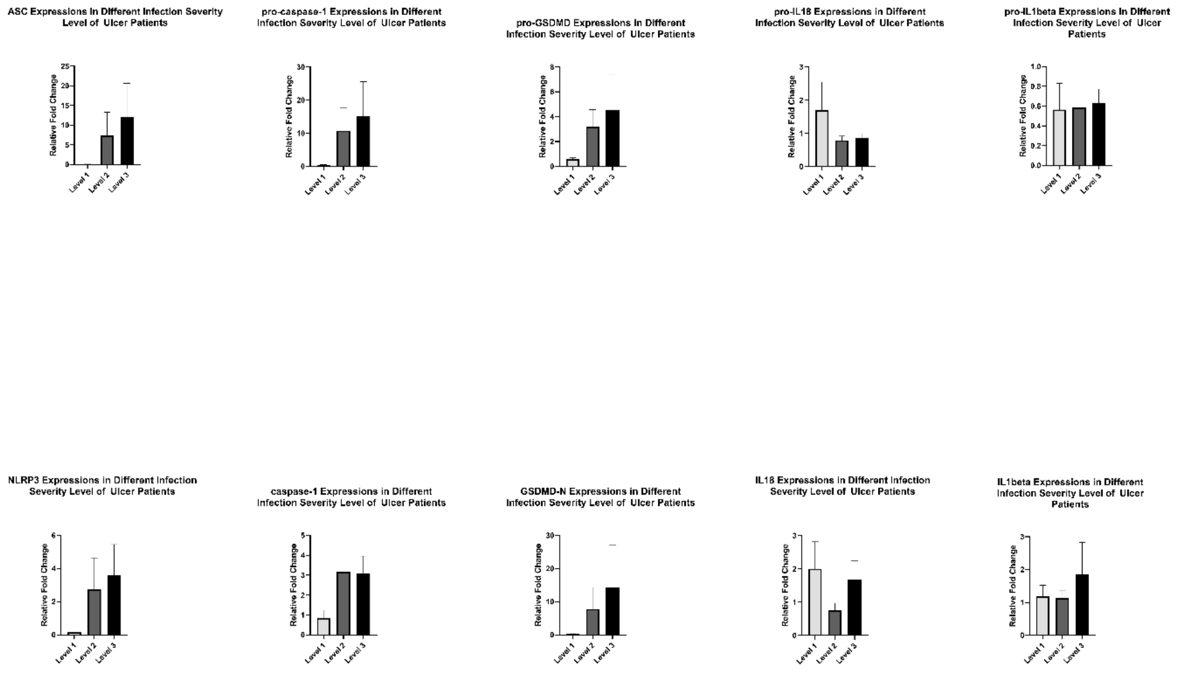

Supplementary Figure S6. Graphs for all patients that show the severity of gastric inflammation and ulceration based on histopathological scoring and included correlation analyses between severity scores and gene expression levels. Error bars in all graphs represent the mean ± standard error of the mean (SEM), unless otherwise indicated.

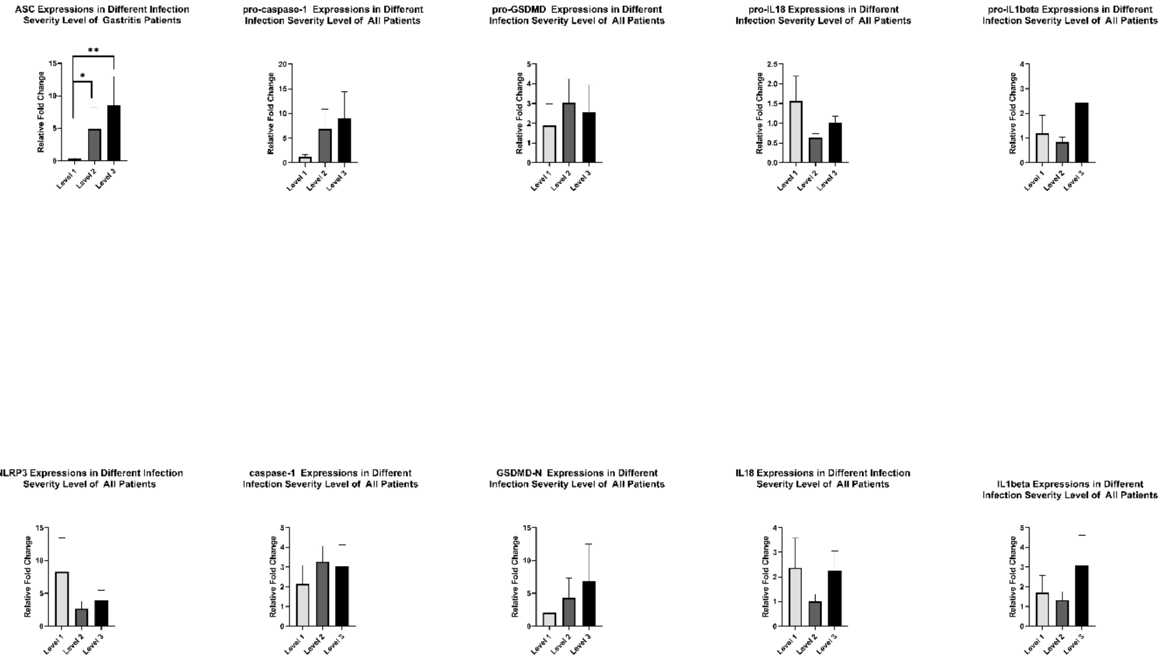

Supplementary Figure S7. Imaging was performed with the ChemiDoc device, and the ImageLab program was used to detect band intensity. When grouping patients, patients with similar protein concentrations isolated from tissues were selected, and care was taken to include patients from the control groups in each group. Patient coding: **N:** uninfected healthy patient, **U:** uninfected ulcer patient, **G:** uninfected gastritis patient, **HpU:** *H. pylori*-infected ulcer patient, **HpG:** *H. pylori*-infected gastritis patient.

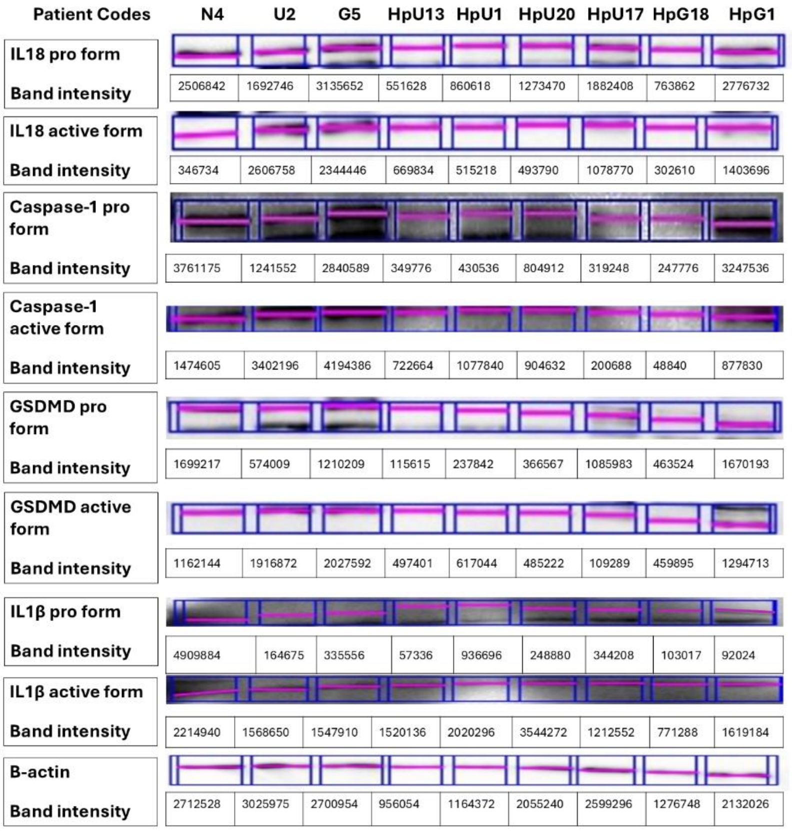

Patient Codes	N4	U2	G5	HpU13	HpU1	HpU20	HpU17	HpG18	HpG1
IL18 pro form – Band intensity	2506842	1692746	3135652	551628	860618	1273470	1882408	763862	2776732
IL18 active form – Band intensity	346734	2606758	2344446	669834	515218	493790	1078770	302610	1403696
Caspase-1 pro form – Band intensity	3761175	1241552	2840589	349776	430536	804912	319248	247776	3247536
Caspase-1 active form – Band intensity	1474605	3402196	4194386	722664	1077840	904632	200688	48840	877830
GSDMD pro form – Band intensity	1699217	574009	1210209	115615	237842	366567	1085983	463524	1670193
GSDMD active form – Band intensity	1162144	1916872	2027592	497401	617044	485222	109289	459895	1294713
IL1β pro form – Band intensity	4909884	164675	335556	57336	936696	248880	344208	103017	92024
IL1β active form – Band intensity	2214940	1568650	1547910	1520136	2020296	3544272	1212552	771288	1619184
B-actin – Band intensity	2712528	3025975	2700954	956054	1164372	2055240	2599296	1276748	2132026

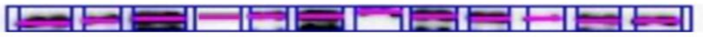
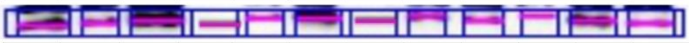
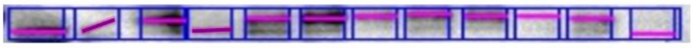
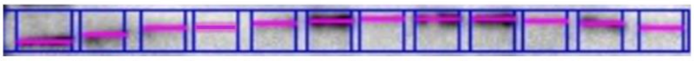
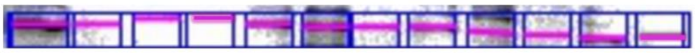
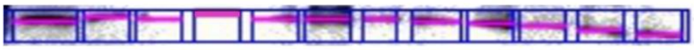
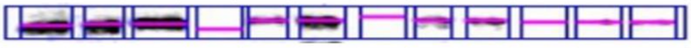
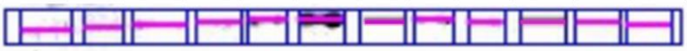
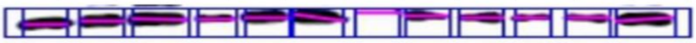

Patient Codes	N5	U3	G2	HpU18	HpU7	HpU8	HpG17	HpG22	HpG3	HpG12	HpG16	HpG13
IL18 pro form Band intensity	4850440	2734320	6484429	112317	2472708	4288095	2977410	3709980	3837353	1074960	3754366	3781080
IL18 active form Band intensity	3284706	1520064	5102482	92024	792276	4940310	113017	2108868	1150383	147936	3858435	1262595
Caspase-1 pro form Band intensity	1258320	134317	1825552	92024	961728	2404192	805648	1016144	1173440	210304	606992	114928
Caspase-1 active form Band intensity	1054760	631760	469424	135017	340800	1704032	352272	1151632	1377552	222864	719360	290144
GSDMD pro form Band intensity	1565127	141027	201952	92013	1224015	1260810	186197	900739	229012	82480	813601	598701
GSDMD active form Band intensity	2565522	146025	231450	16517	1129035	1720880	66790	902730	269715	82680	92024	62017
IL1β pro form Band intensity	1705304	1246024	2839212	113805	612638	1629150	42795	718044	673330	118830	455925	575520
IL1β active form Band intensity	232662	179257	380070	139170	445601	2031645	426124	358748	202152	23313	58395	172545
B-actin Band intensity	6076650	5987640	7017213	3349424	5022765	5706373	513450	3925020	4121760	2723784	3639390	7130380

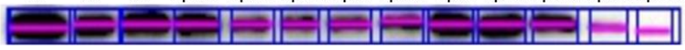
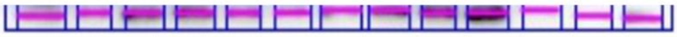
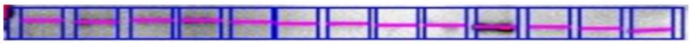
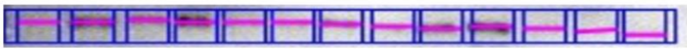
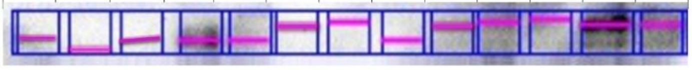
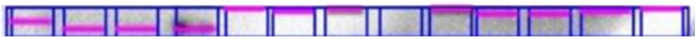
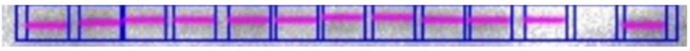
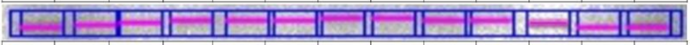
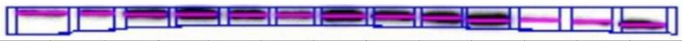

Patient Codes	N2	U4	G4	HpU21	HpU10	HpU13	HpU3	HpU12	HpU5	HpU16	HpG10	HpG15	HpG14
IL18 pro form Band intensity	4542624	4316040	5976474	4612716	3582002	3537180	3983151	3776397	4663782	4755358	5769828	1951905	155022
IL18 active form Band intensity	2688640	1552096	3287600	3275088	1853866	1448895	1556843	3070540	3349066	4961399	632960	423900	1469856
Caspase-1 pro form Band intensity	2445433	1334451	3121331	3134641	1539655	1976779	1565228	3450393	3324431	4100619	609202	410071	909202
Caspase-1 active form Band intensity	396435	702832	521573	803361	386464	344825	475423	192842	376389	2579148	347061	71188	495404
GSDMD pro form Band intensity	243731	180420	231628	2767424	719040	104377	142352	114864	101918	502944	421840	3391960	1545328
GSDMD active form Band intensity	316208	282112	441584	1371248	97168	87536	92024	102718	454359	252944	141328	960920	202720
IL1β pro form Band intensity	698352	693440	580256	663072	736720	434736	787168	830560	968448	954944	185152	463535	1341184
IL1β active form Band intensity	573200	264944	252624	518848	278912	156768	594528	402240	490288	527072	393746	282336	517488
B-actin Band intensity	5040068	3618345	6291054	6397110	4718588	3696032	6205951	5293256	5279537	6531120	1405373	2571046	6265231

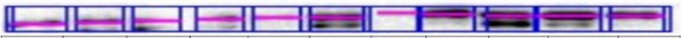
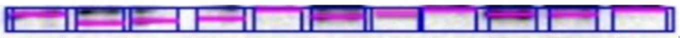
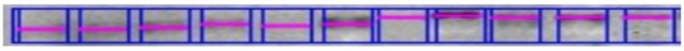
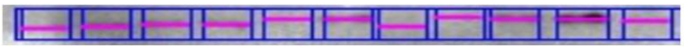
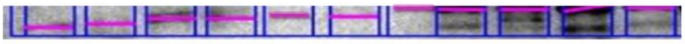
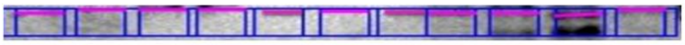
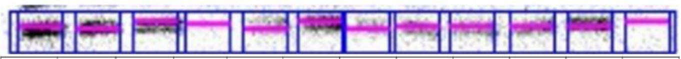
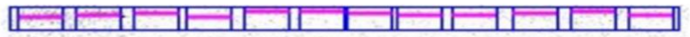
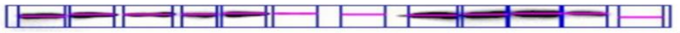

Patient Codes	N1	U1	G1	HpU6	HpU11	HpU2	HpG9	HpG21	HpG8	HpG6	HpG2	
IL18 pro form Band intensity	315571	311175	95625	192390	72975	1033702	95019	1436738	814830	1855500	855831	
IL18 active form Band intensity	301580	94140	120165	36330	15360	425425	92024	47175	681720	225330	30022	
Caspase-1 pro form Band intensity	487408	715440	1064464	799440	282880	1874304	141616	2298128	1053424	1766608	636032	
Caspase-1 active form Band intensity	301808	451456	397616	447184	216144	702192	138880	124560	675680	2192779	213248	
GSDMD pro form Band intensity	466741	315639	968439	720647	138558	137496	55581	3913196	2108442	5053573	2460410	
GSDMD active form Band intensity	335223	89989	266951	149838	38418	2176	8376	519520	304708	713065	293828	
IL1β pro form Band intensity	1616326	1096976	1270036	144483	680085	1223099	566355	792302	792999	756840	1131945	90406
IL1β active form Band intensity	380022	319566	118677	55641	71043	136663	46750	65756	107100	112506	174505	98940
B-actin Band intensity	6849320	7079419	7073073	4723200	70082643	1805969	107521	10133850	8473240	9601240	5471866	47880

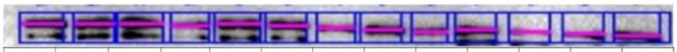
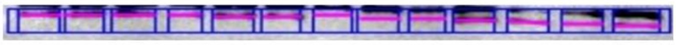
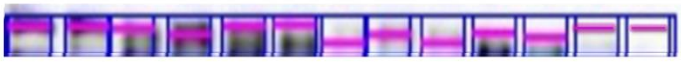
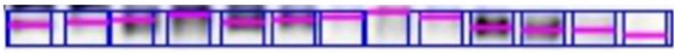
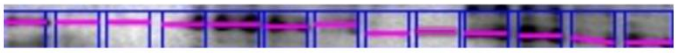
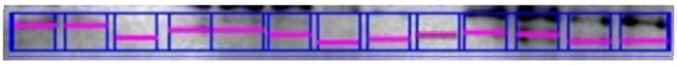
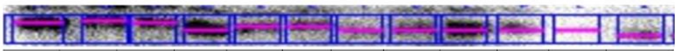
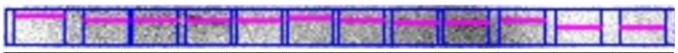
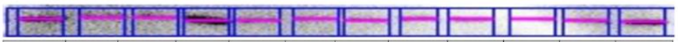

Patient Codes	N3	U5	G3	HpU4	HpU9	HpU14	HpU19	HpU15	HpG11	HpG5	HpG7	HpG19	HpG4
IL18 pro form Band intensity	3790813	3679184	3954348	1596945	3715272	3272880	1720620	3121184	1555260	3001536	669584	1581424	3376314
IL18 active form Band intensity	1200112	1470795	1736635	509964	15737761	249800	45945	130880	153384	1346144	702272	139776	480582
Caspase-1 pro form Band intensity	2220930	1857945	49546528	2836980	763260	196860	503265	1089915	711705	269025	299535	150218	36565
Caspase-1 active form Band intensity	160618	93790	813367	791843	815969	715272	72880	17206	203121	2603001	11424337	63141	20112
GSDMD pro form Band intensity	1421353	90593	233138	1782552	4826934	3012094	1415981	308363	161218	3220837	3166454	1574829	2501065
GSDMD active form Band intensity	116701	715700	269637	890596	99015	35853	143480	84252	92024	555594	10860455	52443	233700
IL1β pro form Band intensity	1869079	1491368	1345542	2709393	493703	319617	188581	302838	2602905	1248166	36006	857873	
IL1β active form Band intensity	81396	110041	89114	194429	106607	39763	49674	82263	153782	116348	16235	59432	
B-actin Band intensity	441643	193987	206329	865742	275706	184875	243882	329672	463777	228310	327811	691356	

The original, uncut full Western blot images are provided below:

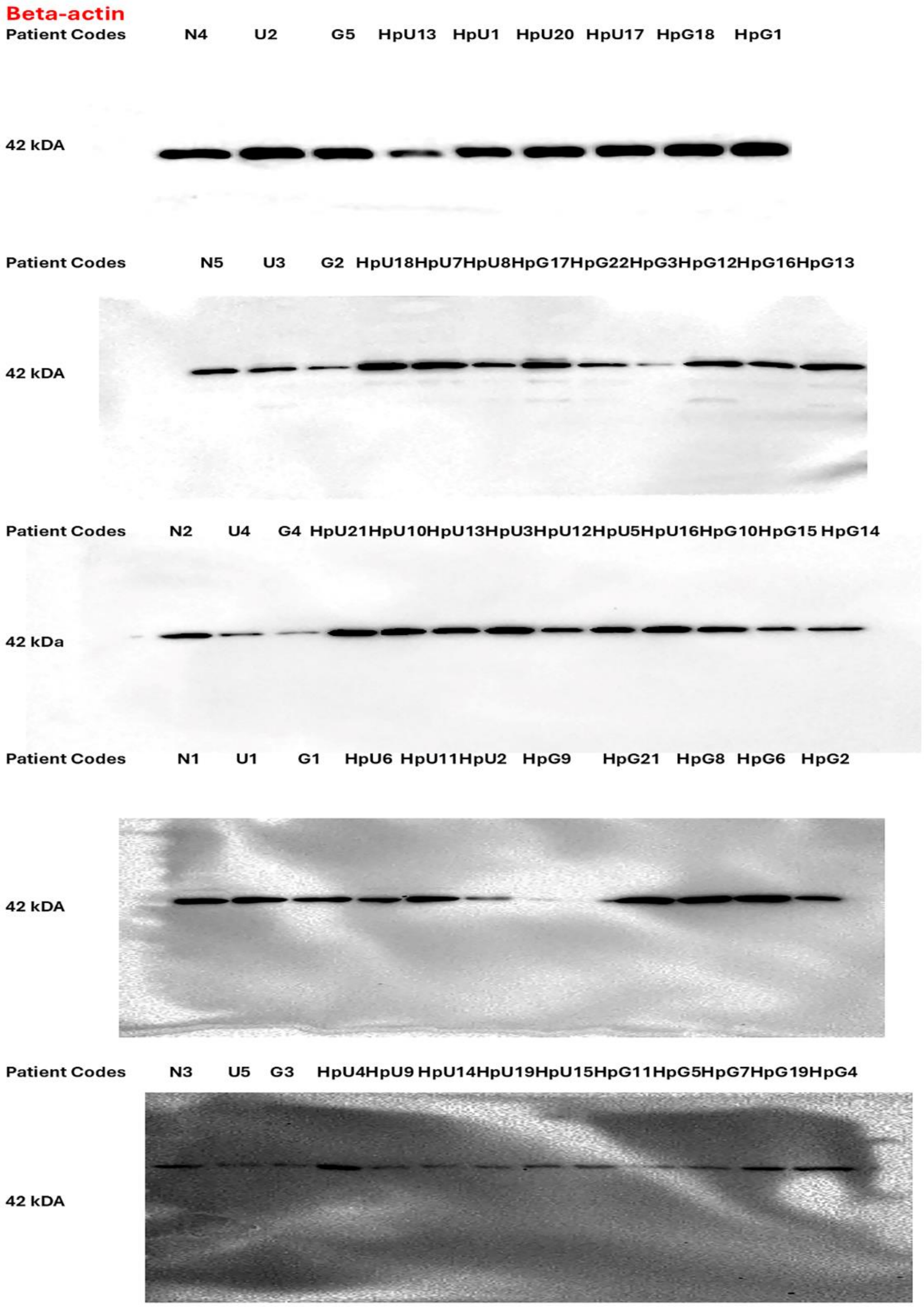

IL-1beta

Patient Codes N4 U2 G5 HpU13HpU1HpU20 HpU17 HpG18 HpG1

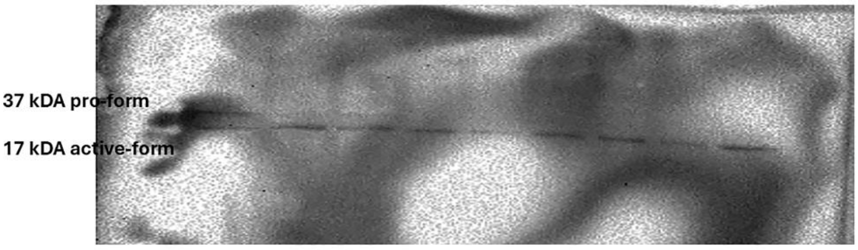

Patient Codes N5 U3 G2 HpU18 HpU7 HpU8 HpG17 HpG22 HpG3HpG12HpG16 HpG13

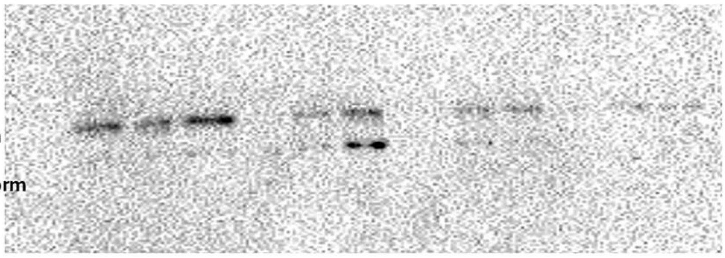

Patient Codes N2 U4 G4 HpU21 HpU10 HpU13 HpU3 HpU12 HpU5 HpU16 HpG10 HpG15 HpG14

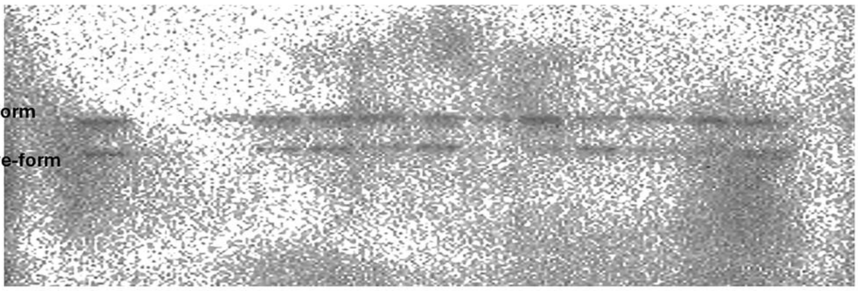

Patient Codes N1 U1 G1 HpU6 HpU11 HpU2 HpG9 HpG21 HpG8 HpG6 HpG2

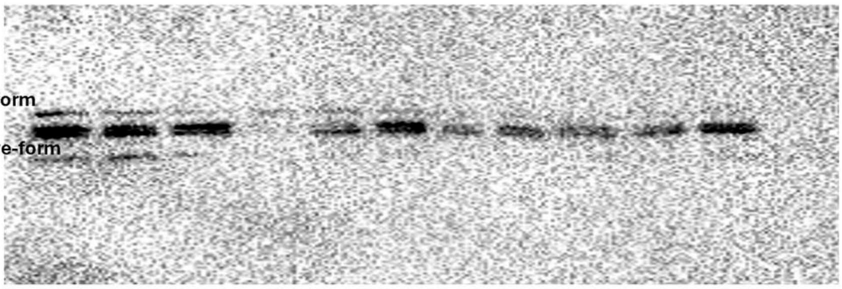

IL-1beta (continue...)

Patient Codes N3 U5 G3 HpU4 HpU9 HpU14 HpU19 HpU15 HpG11 HpG5 HpG7 HpG19 HpG4

37 kDA pro-form
17 kDA active-form

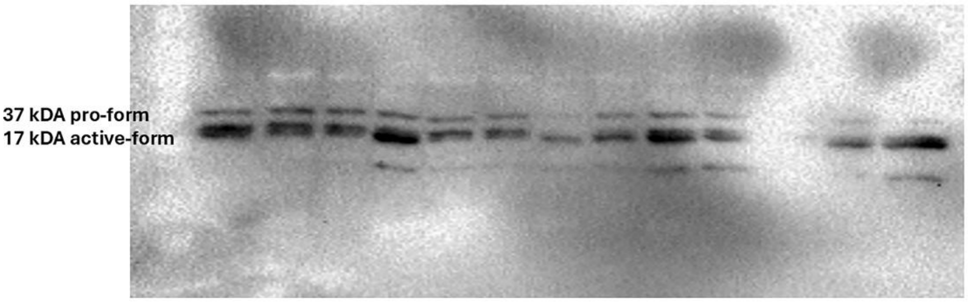

GSDMD

Patient Codes N4 U2 G5 HpU13 HpU1 HpU20 HpU17 HpG18 HpG1

53 kDa GSDMD
30 kDa GSDMD-N

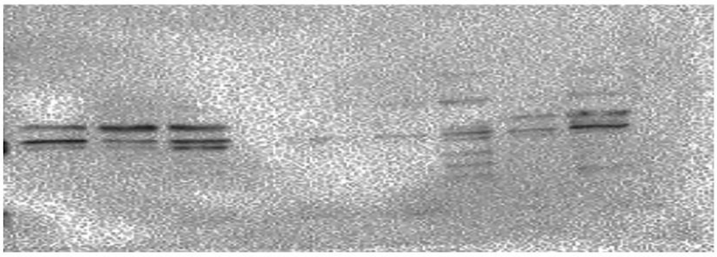

Patient Codes N5 U3 G2 HpU18 HpU7 HpU8 HpG17 HpG22 HpG3 HpG12 HpG16 HpG13

53 kDa GSDMD
30 kDa GSDMD-N

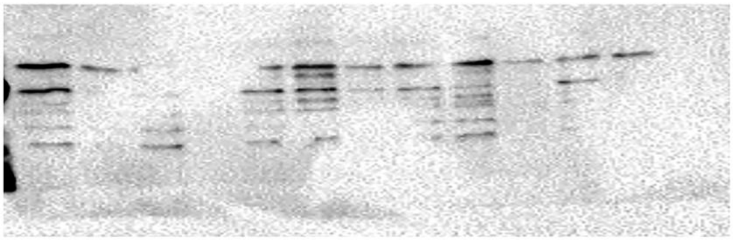

Patient Codes N2 U4 G4 HpU21 HpU10 HpU13 HpU3 HpU12 HpU5 HpU16 HpG10 HpG15 HpG14

53 kDa GSDMD
30 kDa GSDMD-N

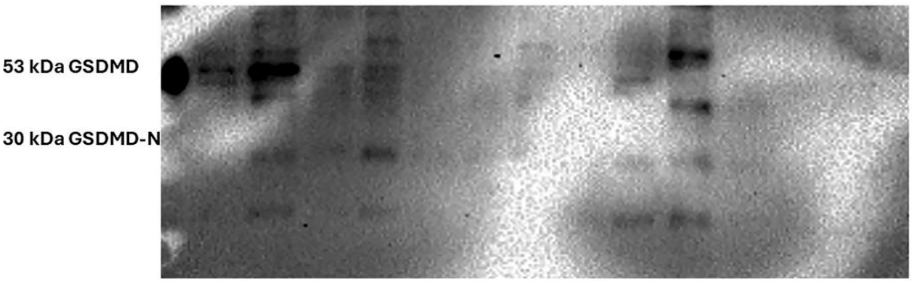

GSDMD (continue...)

Patient Codes N1 U1 G1 HpU6 HpU11 HpU2 HpG9 HpG21 HpG8 HpG6 HpG2

53 kDa GSDMD

30 kDa GSDMD-N

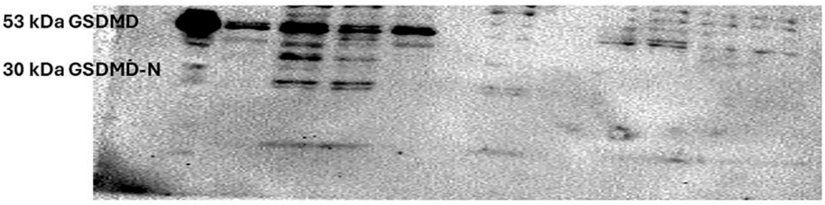

Patient Codes N3 U5 G3 HpU4 HpU9 HpU14 HpU19 HpU15 HpG11 HpG5 HpG7 HpG19 HpG4

53 kDa GSDMD

30 kDa GSDMD-N

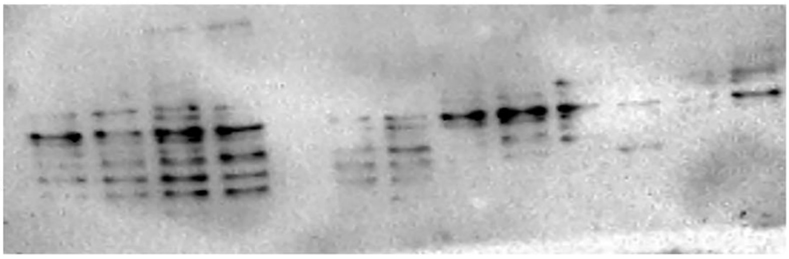

Caspase-1

Patient Codes N4 U2 G5 HpU13 HpU1 HpU20 HpU17 HpG18 HpG1

45 kDA pro-form

23 kDA active-form

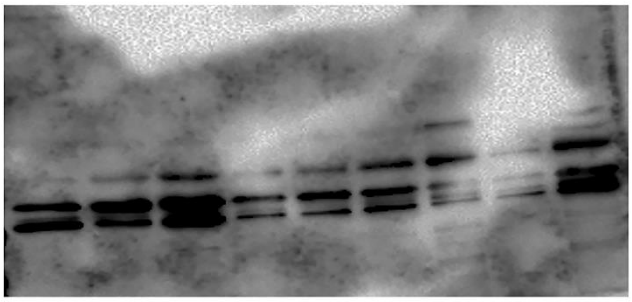

Patient Codes N5 U3 G2 HpU18 HpU7 HpU8 HpG17 HpG22 HpG3 HpG12 HpG16 HpG13

45 kDA pro-form

23 kDA active-form

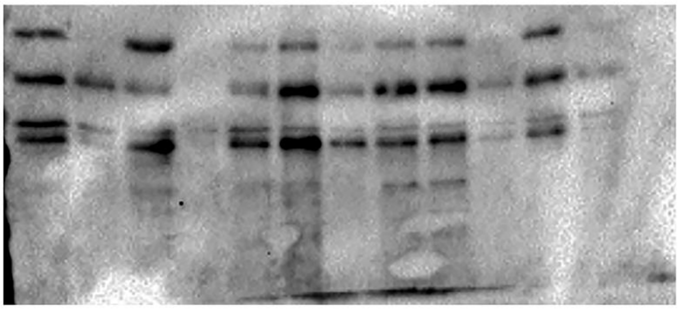

Caspase-1 (continue...)

Patient Codes N2 U4 G4 HpU21 HpU10 HpU13 HpU3 HpU12 HpU5 HpU16 HpG10 HpG15 HpG14

45 kDA pro-form
23 kDA active-form

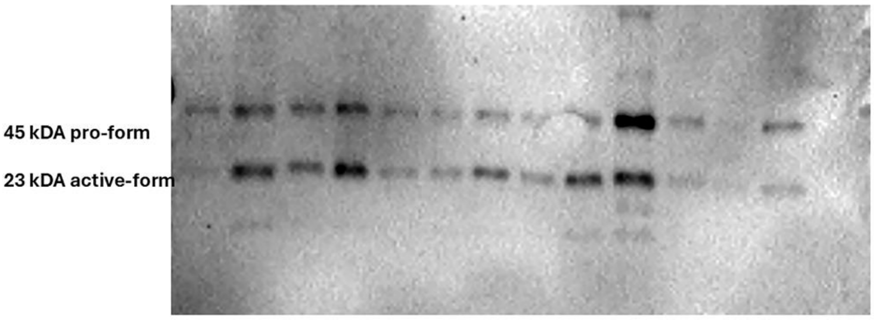

Patient Codes N1 U1 G1 HpU6 HpU11 HpU2 HpG9 HpG21 HpG8 HpG6 HpG2

45 kDA pro-form
23 kDA active-form

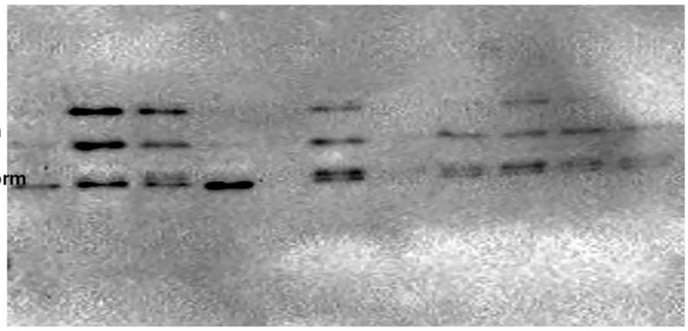

Patient Codes N3 U5 G3 HpU4 HpU9 HpU14 HpU19 HpU15 HpG11 HpG5 HpG7 HpG19 HpG4

45 kDA pro-form
23 kDA active-form

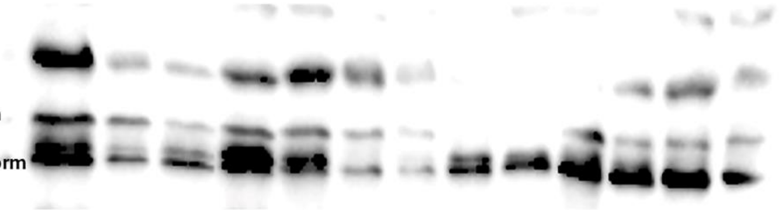

IL-18

Patient Codes N4 U2 G5 HpU13 HpU1 HpU20 HpU17 HpG18 HpG1

24 kDa pro-form
18 kDA active-form

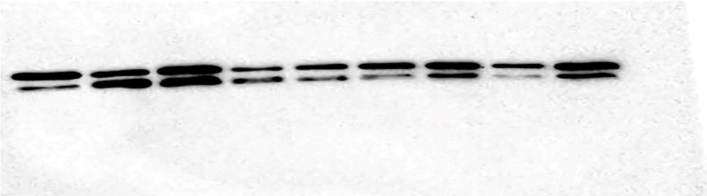

Patient Codes N5 U3 G2 HpU18 HpU7 HpU8 HpG17 HpG22 HpG3 HpG12 HpG16 HpG13

24 kDa pro-form
18 kDA active-form

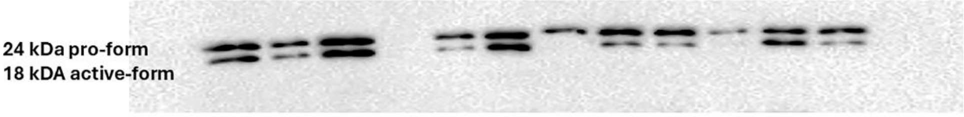

IL-18 (continue...)

Patient Codes N2 U4 G4 HpU21 HpU10 HpU13 HpU3 HpU12 HpU5 HpU16 HpG10 HpG15 HpG14

24 kDa pro-form
18 kDA active-form

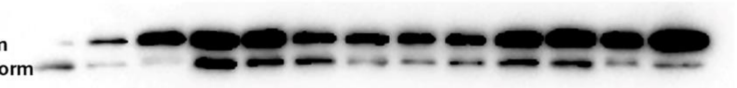

Patient Codes N1 U1 G1 HpU6 HpU11 HpU2 HpG9 HpG21 HpG8 HpG6 HpG2

24 kDa pro-form
18 kDA active-form

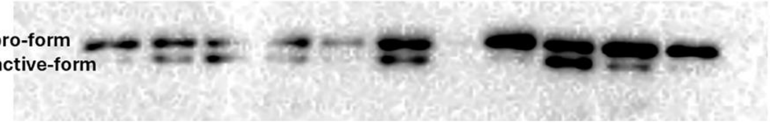

Patient Codes N3 U5 G3 HpU4 HpU9 HpU14 HpU19 HpU15 HpG11 HpG5 HpG7 HpG19 HpG4

24 kDa pro-form
18 kDA active-form

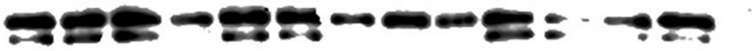

Supplement: Supplementary file 1 [file biology-14-00634-s001.zip › biology-3610212-supplementary.pdf]
